# Is Empathy a “Double-Edged Sword”? How Empathy with Nature Shapes Tourist Well-Being in Wildlife Tourism

**DOI:** 10.3390/bs16101935

**Published:** 2026-10-17

**Authors:** Zhenda Wei, Hong Xu

**Affiliations:** 1 School of History, Culture and Tourism, Liaocheng University, Liaocheng 252000, China; 2 College of Tourism and Service Management, Nankai University, Tianjin 300350, China

**Keywords:** wildlife tourism, empathy with nature, tourist well-being, restorative experience, wildlife anthropomorphism

## Abstract

Empathy is regarded as a key affective driver of individual well-being. In the context of a performance-oriented, hyper-mobile society, the target of empathetic concern has shifted “from humans to nature,” and non-human life is increasingly treated as a new anchoring point onto which emotions are projected and well-being restored. Extant literature, however, usually reduces empathy to a unidimensional emotional amplifier, overlooking the positive–negative bipolarity inherent in empathic responses and the differentiated effects this polarity exerts on well-being. Yet, the gain-and-loss pathways through which empathy with nature influences tourist well-being, together with their boundary conditions, remain unexplored. Adopting an exploratory sequential mixed-methods design, the study deconstructs the mechanism through which empathy with nature affects tourist well-being by means of in-depth interviews and grounded-theory analysis. Three follow-up experiments corroborate the qualitative findings. Results reveal that empathy with nature in wildlife tourism bifurcates into positive and negative strands, exerting asymmetric impacts on tourists’ hedonic and eudaimonic well-being. Restorative experience operates as a vicarious mechanism linking empathy with nature to well-being outcomes. Furthermore, wildlife anthropomorphism asymmetrically moderates these affective pathways—amplifying the harm of negative empathy on hedonic well-being and restoration—while leaving eudaimonic well-being unaffected. Based on mainland Chinese participants in scenario-based zoo encounters, these findings offer an initial, culturally situated account of how empathy with nature shapes tourist well-being in wildlife tourism.

## 1. Introduction

The pursuit of well-being is an innate human capacity, and tourism has become a pivotal channel through which tourists enhance their well-being (Rehman et al., 2025; Yu et al., 2021). Encounters with nature during travel—ranging from immersion in natural landscapes to interactions with wildlife—have been shown to effectively elevate both hedonic and eudaimonic well-being (Ballantyne et al., 2007; J. Chen & Wang, 2022; Xiong et al., 2025). Jia et al. (2026) further demonstrate that nature-based adventure tourism fosters employees’ psychological resilience through meaningful experiences, thereby healing unhappiness. Likewise, immersive wildlife exhibitions have been found to significantly improve tourists’ affect (self-reported well-being gains) and reduce stress (Coolman et al., 2020). In the context of a performance-oriented, hyper-mobile society, individuals in the “achievement society” suffer from chronically compressed time and role overload, eroding the cognitive resources required for interpersonal empathy and engendering collective symptoms of latent loneliness and emotional indifference (B. C. Han, 2015). This deficit in meaning and well-being has triggered a biophilic shift in empathic targets “from humans to nature,” repositioning non-human life—animals, plants, and ecosystems—as novel anchoring points for emotional projection (Fennell, 2024; C. Wang & Lu, 2025). Such a shift not only compensates for the emotional deprivation of nature contact inherent in modern life, but also furnishes tourists with a psychological pathway to generate memorable and well-being experiences within nature-based tourism settings (Farkić et al., 2025).

Empathy is widely assumed to exert a positive influence on well-being, yet no scholarly consensus has been reached. Conceptually, empathy denotes both “the ability to vicariously experience and to understand the affect of other people” and a short-lived psychological process that is cued and elicited in real time by specific situational stimuli (Eisenberg & Fabes, 1990; Tam, 2013). Alarmed by mounting global environmental concerns, Tam (2013) extended the circle of empathy from interpersonal relations to the human-nature interface. Extant research has concentrated on the conservation benefits of empathy with nature for ecosystems and wildlife (L. Wang et al., 2023; C. Wang & Lu, 2025). Although empathy is positively associated with emotional health and well-being (Mehrabian, 2000; Shanafelt et al., 2005), whether individuals actually derive psychosomatic and well-being dividends from empathy with nature remains unexamined and unverified. Conversely, some scholars warn that excessive empathy can erode well-being (Andreychik, 2019; O’Connor et al., 2007). Clarifying the relational pathways and underlying mechanisms linking empathy with nature to tourist well-being is thus an urgent scholarly imperative.

The purpose of this study is to unravel how empathy with nature shapes tourist well-being within the wildlife-tourism context. Wildlife-tourism settings simultaneously broadcast cues of “vitality” and “vulnerability” thereby constituting a high-ecological-validity arena in which tourists’ affective resonance and empathic responses toward nature are markedly intensified (Fennell, 2024; Valença et al., 2021). Despite this promising backdrop, two critical gaps remain. First, the psychological mechanism translating empathy with nature into well-being is still a “black box.” We propose that restorative experience—vicarious relief from mental fatigue through empathic connection with wildlife—serves as the pivotal conduit. Second, the boundary conditions of this process are unknown. Wildlife anthropomorphism, the tendency to ascribe human-like qualities to animals, may amplify or dampen the empathy–well-being link by rendering wildlife more or less psychologically proximate. Accordingly, this study addresses three inter-related questions: (1) Can wildlife encounters elicit empathy with nature and tourist well-being? (2) Does—and through which mechanisms—empathy with nature influence tourist well-being? (3) What boundary conditions govern the above relationship? To answer these questions, we adopt an exploratory sequential mixed-methods design. First, depth interviews and grounded-theory analysis deconstruct the dimensionalities of both empathy with nature and tourist well-being in wildlife settings and explicate how divergent empathy experiences translate into well-being outcomes. Three follow-up experiments then test the posited mechanisms and boundary conditions. The convergent findings affirm that empathy with nature plays a pivotal role in forging tourists’ well-being in wildlife-tourism contexts.

## 2. Literature Review

### 2.1. Wildlife Tourism

Wildlife tourism refers to travel motivated primarily by encounters with non-domesticated animals, either in their natural habitats or in captive settings, encompassing both consumptive and non-consumptive activities (Higginbottom, 2004; Shackley, 1996). Activities span bird-watching, whale-watching, general wildlife safaris, visits to zoos and aquaria, scuba diving to observe marine life, and recreational hunting (Shackley, 1996). As a nature-based form of tourism, it offers tourists direct exposure to—and interaction with—wild animals and their living spaces (Rizzolo, 2023; Sthapit et al., 2024). The contemporary research agenda on wildlife tourism has evolved along two inter-related tracks: wildlife-rights protection and tourist-experience enhancement. On the protection front, scholars and practitioners have promoted habitat conservation, sustainable-tourism protocols, and conservation-education campaigns, thereby improving the welfare of wildlife populations (Charles & Hamid, 2022; Fennell et al., 2023; Hehir et al., 2023; Kline et al., 2023). Simultaneously, efforts to optimise on-site experiences—through personalized services, interpretive technologies, and visitor-engagement innovations—have heightened tourists’ sense of participation and, in turn, their well-being (Methorst et al., 2020; Sthapit et al., 2024; R. Yerbury & Weiler, 2020).

Wildlife tourism is increasingly being linked to the creation of tourist well-being. Through interactions with the spatial environments of wildlife tourism, tourists generate a range of cultural services—such as recreation, education, and health enhancement—that in turn foster identity construction, experiential realization, and capacity development (i.e., dimensions of well-being). Spending time in nature and among wildlife intensifies emotional attachment, which may cultivate empathy, caring, and pro-environmental behavior (Hinds & Sparks, 2008). Several scholars have further argued that contact and engagement with nature and wildlife prompt tourists to re-evaluate their anthropocentric and ecocentric value orientations (Bell et al., 2018; Garland & Fredrickson, 2019). Muloin (1998) demonstrated that whale-watching yields psychological benefits, including stress reduction and improved mental health. Y. Zhang et al. (2022) revealed that cultural ecosystem services co-created by tourists and animals enhance tourist well-being by boosting memorability and perceived authenticity. Collectively, these studies suggest that wildlife tourism possesses the potential to evoke tourists’ emotional experiences and influence their well-being, yet the underlying mechanisms remain to be fully explicated.

### 2.2. Empathy with Nature

Empathy with nature can reshape individuals’ cognitions and affections toward the natural world, forging reciprocal connections that tighten the human–nature bond (Di Fabio & Kenny, 2021; Jing et al., 2022). Mirroring interpersonal empathy, empathy with nature is also construed through a two-dimensional lens comprising cognitive and affective components. Specifically, it denotes the cognitive and affective capacity both to comprehend and to share the experiences of nature (Fido & Richardson, 2019). Cognitive empathy with nature manifests as the ability to recognize and understand natural situations by drawing on prior experience or environmental knowledge, whereas affective empathy with nature entails the identification and emotional contagion elicited by natural entities (A. Perry & Shamay-Tsoory, 2013). Individuals high in empathy with nature are thought to adopt the perspective of nature more readily, to worry about its plight, and to establish stronger affiliations with it (Kandaurova & Lee, 2019). Beyond the cognitive–affective dichotomy, empathy with nature can also be partitioned into state- and trait-based forms. The former—nature-induced empathy—refers to situation-specific empathic concern for a natural target in distress, typically evoked through perspective-taking manipulations (Tam, 2013). The latter—trait empathy with nature—captures a stable disposition to understand and share the experiences of the natural world (Preylo & Arikawa, 2008). In the present study, empathy with nature is conceptualized as a short-lived psychological process triggered when tourists encounter wildlife during wildlife-tourism experiences; it is therefore a nature-induced empathy operating through both affective and cognitive pathways.

Empirical work on empathy with nature remains scarce and is narrowly focused on its antecedents and consequences (Teh et al., 2026; K. Wang et al., 2026). Schultz (2000) examined whether objects that embody egoistic, altruistic, or biospheric values differentially activate individuals’ empathy with nature. Batson (2011) further showed that wildlife anthropomorphism—ascribing human-like qualities to animals and plants—enhances empathic concern for these entities. Consequence-oriented studies have predominantly linked empathy with nature to pro-environmental behavior (Batson et al., 2015; X. Wang et al., 2020). Drawing on psychodynamic theory and the empathy–altruism hypothesis, L. Wang et al. (2023) demonstrated that participants higher in empathy with nature displayed stronger pro-environmental actions, while also documenting the moderating role of self-construal. Collectively, the current research is both limited and fragmented; multi-perspective inquiries are urgently needed to broaden the field.

### 2.3. Tourist Well-Being

Scholarly inquiries into well-being originated in philosophy: more than two millennia ago, thinkers in both Eastern and Western civilizations began to explore the nature of the well-being (Joshanloo, 2014). These reflections eventually crystallized into two dominant philosophical traditions—Hedonism and Eudaimonism (Waterman, 1993). Mirroring this philosophical bifurcation, tourist well-being is routinely parsed into hedonic and eudaimonic dimensions (Smith & Diekmann, 2017). Hedonic well-being denotes the immediate satisfaction of physiological or psychological needs and the resultant pleasurable affect; eudaimonic well-being arises from deep engagement and the full actualization of personal potentials, yielding a sense of self-realization (Ryan & Deci, 2001). Hedonic well-being is closely tied to positive emotion, which is momentary, whereas eudaimonic well-being is anchored in the perceived meaning of activities and therefore more enduring (Huta & Ryan, 2010). Related tourism research repeatedly shows that travel bequeaths both types of well-being to tourists (Sirgy & Uysal, 2016). Jia et al. (2026), for instance, demonstrate that nature-adventure tourism heals employees’ unhappiness through a “meaning–resilience” chain fueled by four sources of meaning—challenging experiences, love for nature, social connection and acceptance of suffering. A comprehensive analysis of tourist well-being must therefore simultaneously embrace its hedonic and eudaimonic facets.

A growing body of research has examined how human–wildlife interactions affect human well-being (Grotto & Buja, 2026; D. J. Perry et al., 2025; White et al., 2023). R. M. Yerbury and Boyd (2019), for instance, analysed encounters with wild dolphins and demonstrated that such experiences enhance well-being through heightened connectivity, affective vitality, and a renewed sense of meaning. On the hedonic side, wildlife tourism reliably generates positive affect—wonder, joy, and excitement—while tourists observe wildlife at close range (Bell et al., 2018; Curtin, 2009; Curtin & Kragh, 2014; McIntosh & Wright, 2017). On the eudaimonic side, the same encounters foster cognitive shifts toward ecocentric values and self-transcendent perspectives that underpin personal growth (Bell et al., 2018; Garland & Fredrickson, 2019). Even indirect activities—volunteering for wildlife monitoring or habitat restoration—imbue tourists with spiritual significance and altruistic fulfilment (Corral-Verdugo, 2012; Van den Born et al., 2018; Vidon et al., 2018). Collectively, these studies confirm that wildlife tourism can simultaneously elevate tourists’ hedonic and eudaimonic well-being, and a few have begun to trace the underlying affective routes. Yet empathy with nature—a significant emotional experience of wildlife encounters—remains strikingly absent from the explanatory framework. Whether and how empathy with nature, elicited in wildlife-tourism settings, translates into well-being gains is therefore a theoretically consequential question.

## 3. Research Design

**Figure 1:**
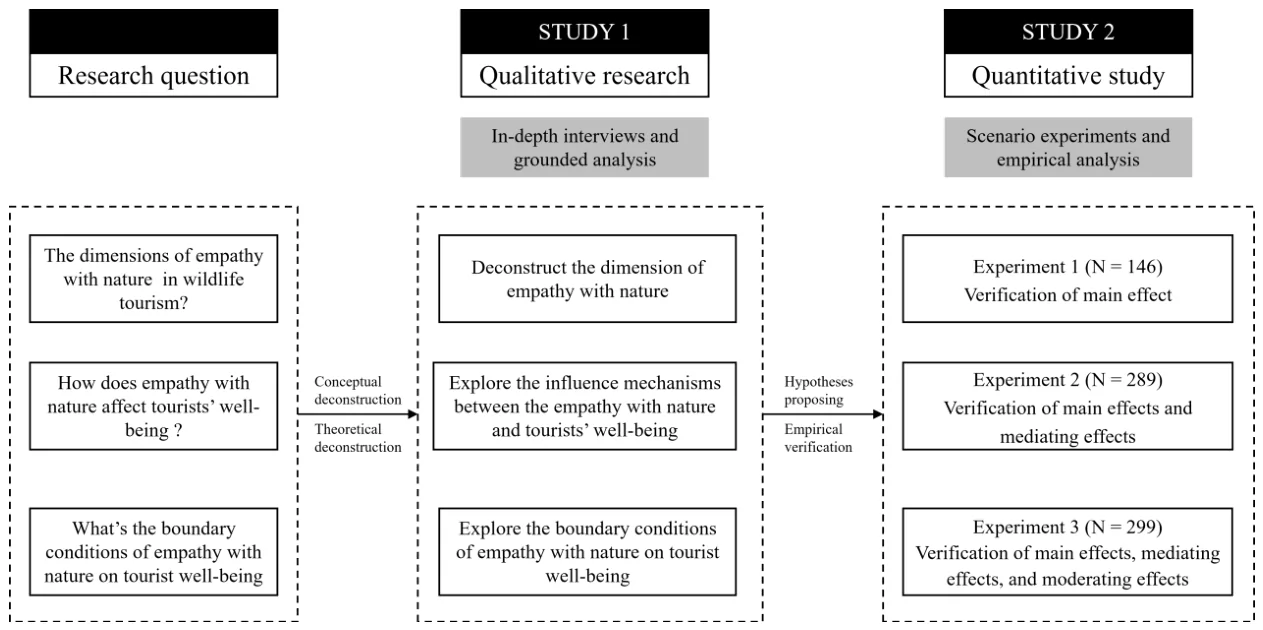
Study design.

To unpack how empathy with nature influences tourist well-being in wildlife-tourism settings, an exploratory sequential mixed-methods design (Figure 1) is employed (Ouerghemmi et al., 2023). Qualitative phase (Study 1). Through semi-structured in-depth interviews and grounded-theory analysis, we first deconstruct the dimensionalities of (a) empathy with nature and (b) tourist well-being as they emerge in wildlife encounters, and explores the influence mechanism and boundary conditions of empathy with nature on hedonic and eudaimonic well-being. Three follow-up quantitative experiments (Study 2) translate the qualitative model into testable hypotheses, operationalise the constructs, and provide causal evidence for the pathways and moderators unearthed in Study 1.

## 4. Study 1: Qualitative Research

Study 1 employs the procedural grounded-theory approach (Strauss & Corbin, 1990) to collect and analyse textual data. We first review the relevant literature to form an initial theoretical lens. Next, drawing on this preliminary work and the study’s practical needs, we develop a interview protocol (Supplementary Material S1) and conduct in-depth interviews with selected participants to obtain primary data. We then proceed with open and axial coding to distil the key elements through which empathy with nature and tourist well-being interact in wildlife-tourism settings. Finally, selective coding is performed to integrate these elements into a coherent theoretical framework that explicates the influence mechanisms and boundary conditions.

### 4.1. Data Collection

Data were gathered through one-on-one in-depth interviews. To ensure sample relevance, theoretical sampling and constant comparison guided both participant selection and sample-size determination, with “theoretical saturation” as the stopping rule: recruitment ceased when additional data produced no new concepts or categories. In practice, three complementary channels were used to capture the widest possible spectrum of wildlife tourists. First, recruitment notices were posted on the social-media platform Xiaohongshu to attract respondents who met the preset criteria. Second, snowball sampling was employed: initial interviewees and the researchers’ personal networks recommended further eligible tourists. Third, convenience sampling on-site was carried out: the researchers visited nationally representative wildlife-tourism destinations to locate and invite suitable candidates. All potential respondents underwent a brief screening to confirm at least one wildlife-tourism experience within the past year. Before each interview, the purpose and voluntary nature of the study were explained, and confidentiality assurances were provided so that participants felt comfortable sharing authentic experience. After the completion of the interview, according to the quality of the interview and the richness of the collected information, the participants were given corresponding compensation ($5.55–$8.33). In total, 30 interviews were conducted. After the 26th interview no new insights emerged; four additional conversations confirmed this saturation. Participants ranged from 18 to 53 years of age, 33.3% male and 66.7% female. Twenty-four transcripts were randomly selected for open, axial, and selective coding to build the theoretical model. The remaining six transcripts were subsequently re-coded; no fresh categories appeared, indicating that theoretical saturation had been achieved and the findings are both reliable and valid.

### 4.2. Data Analysis

All transcripts were imported into NVivo. Following the classic open-coding logic, each sentence was read line-by-line and any excerpt that referred to tourists’ emotional experiences or to the antecedents/consequences of tourist well-being was assigned a free node. This first sweep produced 215 raw labels that faithfully summarised the original statements. Because these labels overlapped, repeated, or occasionally misrepresented meaning, the authors met repeatedly to compare, collapse, and refine them. Through iterative discussion and constant comparison the pool was reduced to 53 distinct initial concepts, which were then grouped into 19 preliminary categories that captured the range of empirical phenomena identified in the interviews.

Next, axial coding was performed to interrogate the 19 preliminary categories generated during open coding. Through constant comparison and theoretical clustering, these categories were distilled into eight sub-dimensions: positive empathy with wildlife, negative empathy with wildlife, wildlife affective recognition, hedonic well-being, eudaimonic well-being, restorative experience, tourist traits, and wildlife anthropomorphism. Drawing on prior work (Larionow, 2025; Wu et al., 2025), the first three sub-dimensions were collapsed under the higher-order construct “empathy with nature,” while hedonic and eudaimonic well-being were treated as two facets of the second-order construct “tourist well-being.” The remaining three sub-dimensions—restorative experience, tourist traits, and wildlife anthropomorphism—were retained as separate core categories. Thus, the eight sub-dimensions were ultimately reorganised into five main categories: empathy with nature, tourist well-being, restorative experience, tourist traits, and wildlife anthropomorphism.

Selective coding followed to identify a central category capable of integrating the five main categories into a coherent storyline. This phase demanded a higher level of abstraction and theoretical synthesis (Glaser & Strauss, 2017). Through the in-depth analysis of the initial concepts, initial categories and main categories obtained by open coding and axial coding, the relatively clear relationship between the main categories is gradually emerging. Based on the research theme, “How does natural empathy affect the well-being of wildlife tourists” is set as the core category. Among them, empathy with nature subsumes both positive and negative valences toward wildlife, whereas tourist well-being comprises hedonic and eudaimonic sub-dimensions; restorative experience, tourist traits, and wildlife anthropomorphism operate as linking mechanisms or boundary conditions that shape the strength and direction of the core relationship.

### 4.3. Qualitative Summary

The grounded-theory phase furnishes an evidential base for delineating how empathy with nature relates to tourist well-being and supplies the conceptual groundwork for subsequent hypothesis-testing. Three insights are central. First, tourists routinely read emotional states in wildlife. In general, tourists are more likely to identify the emotions of mammals or primates than other types of animals, which may be related to the similarity between wild animals and humans (Westbury & Neumann, 2008). These readings split into positive and negative valences. When animals are viewed in enriched, stress-free settings tourists catch their positive affect, raising their own hedonic tone; conversely, perceived distress in animals triggers negative empathy, lowering mood and momentary well-being. Second, empathy with nature is itself restorative. Consistent with clinical evidence that human–animal contact reduces cortisol and elevates oxytocin (Odendaal & Lehmann, 2000), wildlife tourists report relief from anxiety and mental fatigue, an effect they describe as vicarious—“I felt healed through feeling for them.” Third, wildlife anthropomorphism intensifies the effect: the more tourists attribute sentience and feeling to wildlife, the more reluctant they are to harm them (Gray et al., 2007) and the more positive affect they extract from empathic connection (Clayton et al., 2009). These inductively derived propositions—(1) positive versus negative empathy with nature, (2) restorative experience as mediator, and (3) wildlife anthropomorphism as moderator—are formalized and tested in Study 2.

## 5. Study 2: Experimental Investigation

### 5.1. Theoretical Foundations and Hypothesis Development

#### 5.1.1. Broaden-and-Build Theory

Proposed by Fredrickson (2001), the Broaden-and-Build theory focuses on the positive effects of positive emotions on individuals’ psychology and behavior. The theory posits that, unlike negative emotions that typically evoke narrow action tendencies (e.g., flight), positive emotions broaden people’s scope of attention and cognition, encouraging exploratory, creative, and inclusive thoughts and actions. Individuals experiencing positive emotions are more likely to see the “big picture,” explore novel ideas and experiences, and build social connections with others. Over time, this broadening effect helps individuals accumulate enduring personal resources (Fredrickson & Branigan, 2005)—such as physical, psychological, and social resources—that can be drawn upon in future challenges, providing resilience and protection, and enhancing overall well-being and life satisfaction. The theory has been widely applied in positive psychology, particularly in research on well-being, positive emotions, and interpersonal relationships.

In tourism research, the Broaden-and-Build theory is also frequently invoked to explain how experiences can be enhanced and tourist well-being improved. Y. Chen et al. (2021) found that subjective well-being positively influences tourists’ sharing of travel experiences on mobile social media, thereby increasing their enjoyment of digital travel experiences. Liu et al. (2024) used the theory to show that trait gratitude, well-being, and shared citizenship behaviors are positively interrelated, offering practical insights for safeguarding the well-being of P2P-accommodation micro-entrepreneurs and their guests. However, existing applications of the theory in tourism have focused almost exclusively on positive affect, leaving the developmental trajectory of negative emotions within the broaden-and-build framework largely unexplored (Lundin et al., 2023; Su et al., 2022).

In wildlife tourism settings, observing wildlife in a visibly positive state can evoke positive empathy with nature. According to the broaden-and-build theory, the positive affect generated by such empathic experiences widens individuals’ momentary thought-action scope, producing greater pleasure and satisfaction and thereby raising hedonic well-being (Fredrickson, 2001). Simultaneously, positive empathy helps accumulate enduring personal resources: it strengthens tourists’ emotional bonds with wildlife, heightens perceived care and responsibility toward the animals, and consequently boosts eudaimonic well-being. Conversely, negative empathy is usually linked to avoidance tendencies that constrict opportunities for new actions, ideas, and social connections, reducing the stock of psychological and social resources and limiting gains in both hedonic and eudaimonic well-being (Cosmides & Tooby, 2000). As one respondent noted, “Whether the wildlife receives adequate care and can live happily obviously influences visitors’ feelings. If we buy a ticket and find the animals confined and listless, our mood instantly drops from up to down.(T03)” Therefore, we propose:

**H1.**  *Empathy with nature will influence their well-being—H1a (hedonic well-being) and H1b (eudaimonic well-being). Positive empathy with nature will enhance well-being, whereas negative empathy with nature will diminish it.*

#### 5.1.2. Vicarious Experience Theory

Rooted in Freudian psychoanalysis, vicarious-experience theory posits that personality is structured by three interacting agencies: the id, the ego, and the superego (Freud, 1989). The id operates on the pleasure principle and represents primal needs; the superego internalises societal morality and can inhibit the id; the ego mediates between the two, obeying the reality principle. Psychic functioning therefore requires the ego to regulate the id’s tensions and frustrations, allowing instinctual desires to be displaced rather than directly gratified. When direct access to the original desired object is blocked, the individual seeks a substitute object that approximates the original. Successfully “investing” instinctual energy in this substitute reduces anxiety and restores psychic equilibrium (Hall, 2016). Because the process is one of successive compromise and sublimation, the closer the substitute is to the original object, the greater the felt satisfaction; hence people instinctively prefer the most proximate vicarious fulfilment available (Huang et al., 2015; McKeever, 2015).

Interact with nature is known to generate restorative experiences that replenish directed attention and reduce stress (Kaplan et al., 1989). Emerging evidence shows that empathic contact with animals likewise alleviates anxiety and tension: Sung and Han (2023) found that cognitive and affective empathy toward pets can substitute for human companionship and elevate life satisfaction. In wildlife-tourism settings, positive empathy with nature allows visitors to “catch” the apparent positive affect of free-ranging animals, thereby experiencing pleasant feelings themselves. According to vicarious experience theory, such responses reflect a displacement of the individual’s own desires for freedom, relaxation, and joy onto the observed animals; by empathically sharing the animals’ perceived states, tourists obtain indirect gratification (Freud, 2015). When wildlife appear happy, authentic, and carefree, visitors feel consoled and acquire a sense of vicarious satisfaction. By imagining themselves inside the animals’ relaxed milieu, tourists momentarily partake in that ease, gaining physical and mental restoration. One interviewee expressed this succinctly: “Especially when I sense their happiness—just lying there basking in the sun—while I’m stuck in a 996 schedule, watching them eat and laze around makes me happy; I even wish I could be reincarnated as a panda (T01).” Therefore, we propose:

**H2.**  *Empathy with nature will influence tourists’ restorative experience. Positive empathy with nature will enhance restorative experience, whereas negative empathy with nature will diminish it.*

To integrate these two theoretical lenses, we posit that Broaden-and-Build Theory and Vicarious Experience Theory operate at complementary levels of analysis. Broaden-and-Build Theory provides the overarching functional framework: it predicts whether empathy expands or contracts well-being. Vicarious Experience Theory rooted in psychodynamic theory, provides the specific mechanistic content: it explains how empathy achieves this expansion or contraction through restorative experience. Specifically, restorative experience can be understood simultaneously as (a) the product of vicarious displacement Vicarious Experience Theory and (b) a form of enduring psychological resource that positive empathy helps accumulate Broaden-and-Build Theory. This integrated view allows us to hypothesize that empathy with nature influences well-being both directly (via broadening/building) and indirectly (via restorative experience as vicarious healing).

**H3.**  *Tourists’ restorative experience mediates the relationship between empathy with nature and tourist well-being (H3a: hedonic well-being; H3b: eudaimonic well-being). Restorative experience will transmit a positive effect when empathy is positive and a negative effect when empathy is negative.*

#### 5.1.3. The Moderating Role of Wildlife Anthropomorphism

Epley et al. (2008) define anthropomorphism as the tendency to attribute human-like characteristics, intentions, motives, or emotions to non-human agents. Guthrie (1995) views it as a cognitive strategy for making sense of the world, and instances of wildlife anthropomorphism are commonplace in daily life—for example, the “Mother Earth” trope widely employed by environmental organizations in their campaigns. To date, empirical applications of anthropomorphism have concentrated largely on consumer psychology, human–computer interaction, and environmental psychology. Within tourism studies, the concept has been explored primarily in the contexts of human–robot interaction and destination-brand marketing (B. Han et al., 2020; Li et al., 2024; Ma et al., 2023). More recently, anthropomorphism has gained traction in wildlife-conservation research, where it is regarded as a valuable tool for fostering protection motives by transforming non-human entities into moral subjects worthy of respect (Root-Bernstein et al., 2013). Previous findings indicate that individuals who perceive non-human entities as possessing senses and feelings are less willing to harm them, and that belief in nature’s capacity for emotion increases people’s propensity to care for it (Clayton et al., 2009; Gray et al., 2007).

**Figure 2:**
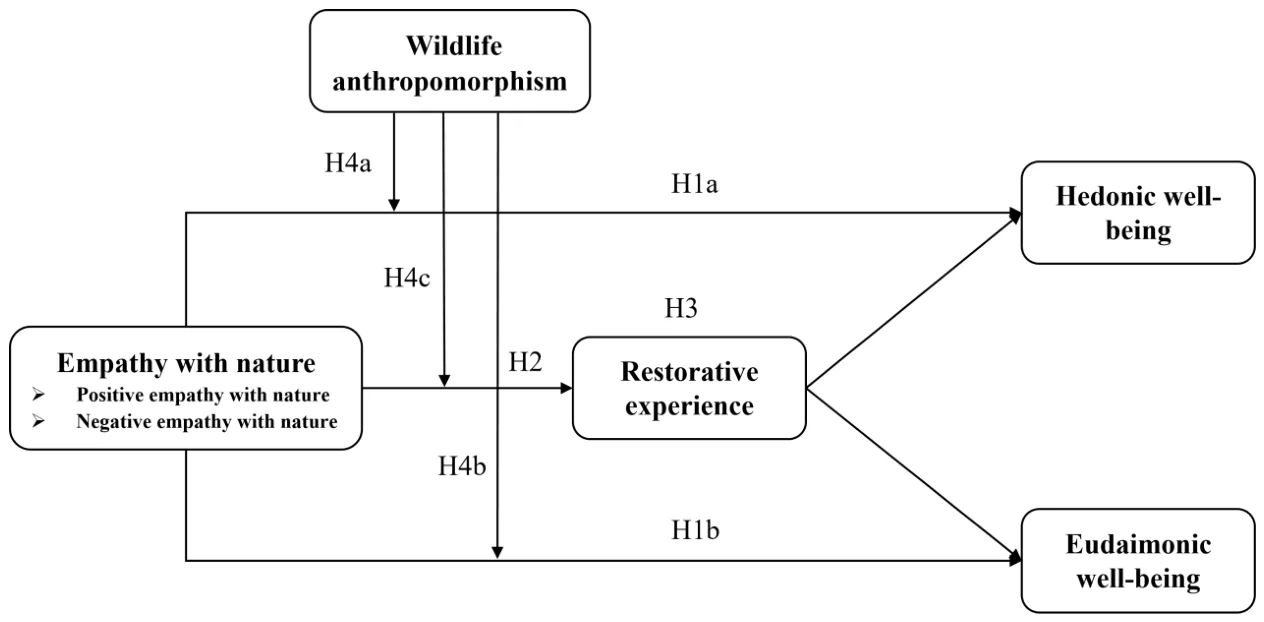
Theoretical Framework.

The relationship between anthropomorphism and empathy is a hot topic in academia. Many studies show that higher levels of anthropomorphism significantly enhance empathy. Yue et al. (2021) found that anthropomorphic materials increase participants’ empathy and reduce their intention to purchase wildlife products. Liu et al. (2024) further demonstrated that anthropomorphic interpretation cues influence tourists’ civil behavior intentions through the mediating role of meaningful experiences. Collectively, these findings suggest that anthropomorphism can amplify empathy and, in turn, restorative outcomes and well-being: the more human-like the target, the stronger the empathic response and the greater the emotional support and meaning derived. We theorize that anthropomorphism operates through two distinct pathways. For hedonic well-being and restorative experience, anthropomorphism functions as an “affective amplifier”: based on Vicarious Experience Theory, the closer the substitute (i.e., a human-like animal) is to the self, the more intense the vicarious emotional experience (McKeever, 2015). Thus, high anthropomorphism simultaneously intensifies the positive vicarious joy derived from positive empathy and the negative vicarious distress derived from negative empathy. For eudaimonic well-being, however, we propose a different prediction. Eudaimonia arises primarily through cognitive value reconstruction—reflecting on animal welfare, ecological ethics, and one’s own life meaning—rather than through surface-level affective contagion. Because our anthropomorphism manipulation conveys only lightweight, surface-level human-like cues (e.g., names, personalities), it is theorized to be insufficient to penetrate the cognitive depth required for meaning-based well-being. Therefore, we do not predict a moderating effect of anthropomorphism on eudaimonic well-being. Therefore, we propose:

**H4.**  *Wildlife anthropomorphism moderates the relationships between empathy with nature and hedonic well-being (H4a) and restorative experience (H4c), but not eudaimonic well-being (H4b) (Figure 2).*

### 5.2. Research Design of Experiment

To test the four hypotheses (Figure 2), we conducted three complementary sub-studies (Table 1). Experiment 1 examines whether empathy with nature affects tourist well-being. Using a between-subjects online experiment, participants were randomly assigned to either a positive-empathy or negative-empathy condition to test the main effect (H1). Experiment 2 compares the well-being consequences of empathy with nature with a no-empathy control and investigates the mediating role of restorative experience. A three-condition between-subjects online experiment (positive empathy vs. negative empathy vs. no empathy) was employed to test both direct (H1, H2) and indirect effects (H3). Experiment 3 maps the boundary conditions of these effects by introducing wildlife anthropomorphism as a moderator. A 3 (empathy with nature: positive vs. negative vs. no empathy) × 2 (wildlife anthropomorphism: high vs. low) between-subjects online experiment was conducted to simultaneously test main, mediating, and moderating effects (H1–H4).

### 5.3. Experiment 1

#### 5.3.1. Purpose

Experiment 1 tests H1. To examine whether empathy with nature influences tourists’ well-being, we constructed a simulated wildlife-tourism scenario designed to elicit either positive or negative empathy with nature and then measured participants’ hedonic and eudaimonic well-being. We predicted that tourists who experience positive empathy with wildlife would report higher well-being than those who experience negative empathy.

#### 5.3.2. Procedure and Materials

Experiment 1 employed a between-subjects online design. Following established scenario-induction protocols (Graziano et al., 2007), we constructed two brief narrative vignettes of equal length that portrayed wildlife in either a positive or negative state. Text-based primes are widely used to activate empathy in tourism research (Sassenrath et al., 2016; Soyoren & Aktas, 2024). At the outset, participants were instructed to imagine themselves on a wildlife-watching trip and were randomly assigned to read one of the two vignettes. Immediately afterward they completed a five-point Likert scale (1 = strongly disagree, 5 = strongly agree) assessing empathy with nature—eight positive items and eight negative items adapted from Andreychik and Migliaccio (2015) and Tam (2013)—as well as hedonic well-being (six items) and eudaimonic well-being (six items), with all well-being items taken from Park and Ahn (2022). A realism-check item (“Could this happen in real life?” 1 = completely impossible, 5 = very likely) was included to verify scenario realism (Liao, 2007), and finally basic demographic information was collected.

#### 5.3.3. Pilot Test

To verify that the scenarios effectively activated empathy with nature, we conducted a pilot study. One hundred participants (66 female; M_age = 31.62, SD = 7.20) were recruited via the Credamo platform—a professional Chinese online survey platform comparable to Amazon Mechanical Turk or Prolific—and randomly assigned to read either the positive- or negative-empathy scenario (Jia et al., 2026). They then completed the empathy with nature and well-being scales used in the main experiment and rated scenario realism.

Mean realism was 4.18 (scale 1–5), indicating high credibility. Participants in the positive-empathy condition reported strong positive empathy M = 3.90, whereas those in the negative-empathy condition reported strong negative empathy M = 4.20. Both values exceed the scale midpoint, confirming successful manipulation. Reliability analyses also showed acceptable internal consistency for all scales, so the materials were retained for the formal experiment.

#### 5.3.4. Formal Experiment

One hundred and sixty participants were recruited through Credamo, a professional Chinese survey platform; after excluding 14 who failed attention checks, 146 valid responses remained (95 female; M_age = 30.08, SD = 6.69). Table 2 reports descriptive statistics and reliabilities for all measures. A priori power analysis using G*Power 3.1 indicated a minimum sample of 126 (ANOVA, two groups, effect size f = 0.25, α= 0.05, power = 0.80), so the obtained sample was adequate. Realism check: the scenario was rated realistic (M = 4.28, 1–5 scale). Positive-empathy participants reported strong positive empathy M= 4.32, negative-empathy participants reported strong negative empathy M = 4.45, confirming successful manipulation. As a cross-valence check, participants in the positive-empathy condition scored substantially lower on the negative-empathy scale than those in the negative-empathy condition (M = 3.39, SD = 0.55 vs. M = 4.45, SD = 0.24; Welch’s t(96.08) = 14.94, *p* < 0.001), and mirror-image patterns held for the positive-empathy scale. One-way ANOVA showed that positive-empathy tourists reported significantly higher hedonic well-being than negative-empathy tourists (M_positive_ = 4.21, SD = 0.53 vs. M_negative_ = 2.29, SD = 1.00; F(1,144) = 209.9, *p* < 0.001, *η*^2^*_p_* = 0.593) and significantly higher eudaimonic well-being (M_positive_ = 4.13, SD = 0.59 vs. M_negative_ = 2.90, SD = 1.08; F(1,144) = 72.8, *p* < 0.001, *η*^2^*_p_* = 0.336). Thus H1 was supported. Regression analyses further indicated that gender and age did not significantly influence either well-being dimension (*p* > 0.05), ruling out demographic confounds.

### 5.4. Experiment 2

#### 5.4.1. Purpose

Experiment 2 tests H1, H2, and H3. By constructing a simulated wildlife-tourism scenario that induces positive empathy, negative empathy, or no empathy toward wildlife, we examine whether empathy with nature influences tourist well-being and whether restorative experience mediates this relationship. We expect positive empathy to generate the highest levels of restorative experience and well-being, followed by the no-empathy condition, while negative empathy will produce the lowest well-being.

#### 5.4.2. Procedure and Materials

Experiment 2 employed a one-factor, three-condition between-subjects online design. To verify that the observed effects are genuinely empathy-driven, a no-empathy control was added. Following the scenario-induction protocol of Graziano et al. (2007), we constructed three narrative scenarios of equal length that portrayed wildlife as positive empathy, negative empathy, or no empathy. To enhance external validity, each text was paired with two colour photographs of the depicted animals; prior studies show that image-plus-text stimuli reliably activate empathic states (Shen et al., 2023; L. Wang et al., 2023).

At the start, participants were instructed to imagine themselves on a wildlife-watching trip and were randomly assigned to read one vignette. Immediately afterward they completed the same 5-point Likert scales used in Experiment 1 for empathy with nature (positive and negative sub-scales), hedonic well-being (6 items), and eudaimonic well-being (6 items). Restorative experience was now measured with the 6-item ROS scale developed by Korpela et al. (2008). Manipulation-check and realism items followed the identical wording and 5-point format employed in Experiment 1, after which basic demographic data were collected.

Data were analyzed with one-way ANCOVAs (covariates: gender, age) for the omnibus tests. Pairwise comparisons were computed on estimated marginal means from the ANCOVA model (i.e., adjusted for the covariates), using the least significant difference (LSD) method (no adjustment); t values were obtained as the mean difference divided by its standard error, with degrees of freedom equal to the model’s error degrees of freedom (284 in Experiment 2 and 291 in Experiment 3). The same analytic strategy was applied in Experiment 3.

#### 5.4.3. Pilot Test

To verify that the vignettes again successfully activated empathy with nature, we recruited 88 participants on Credamo (64 female; M_age = 31.24, SD = 7.14) and randomly assigned them to one of the three text-and-photo conditions. After reading the material, they completed the empathy, restorative-experience, and well-being scales; realism and demographics were also recorded. Mean realism was 4.44, confirming credibility. Independent-samples *t*-tests showed that the positive-empathy group reported significantly higher positive empathy than the none-empathy group (M_positive_ = 4.29, SD = 0.37 vs. M_none_ = 3.76, SD = 0.89; t(57) = 2.98, *p* < 0.01), while the negative-empathy group reported significantly higher negative empathy than the none-empathy group (M_negative_ = 4.41, SD = 0.36 vs. M_none_ = 2.88, SD = 0.84; t(57) = 9.02, *p* < 0.001). These results indicate successful manipulation. Reliability analyses again showed high internal consistency for all scales, so the materials were retained for the formal experiment.

#### 5.4.4. Formal Experiment

Three hundred and forty-one participants were recruited through Credamo; after excluding forty-one who failed attention checks and eleven who had neither traveled nor had a wildlife tourism in the past year, 289 valid responses remained (200 female; M_age_ = 31.45, SD = 6.01). A priori power analysis indicated a minimum sample of 159 (one-way ANOVA, three groups, effect size f = 0.25, α = 0.05, power = 0.80), so the obtained sample was adequate. Realism check: the scenario was rated credible (M = 4.43). Independent-samples *t*-tests showed that the positive-empathy group reported significantly higher positive empathy than the none-empathy group (M_positive_ = 4.26, SD = 0.37 vs. M_none_ = 3.56, SD = 0.84, *p* < 0.001), while the negative-empathy group reported significantly higher negative empathy than the none-empathy group (M_negative_ = 4.46, SD = 0.24 vs. M_none_ = 2.67, SD = 0.68, *p* < 0.001), confirming successful manipulation. As a cross-valence check, participants in the positive-empathy condition also reported somewhat elevated negative empathy relative to the no-empathy group (M = 3.26 vs. 2.67; Welch’s t(171.21) = 6.84, *p* < 0.001), reflecting the mild mixed affect that the positive vignette elicits; their negative-empathy scores nevertheless remained far below those in the negative-empathy condition (M = 4.46).

Tourist well-being. One-way ANCOVAs (controlling for gender and age) revealed a significant effect of empathy with nature on both hedonic (F(2,284) = 768.9, *p* < 0.001, *η*^2^*_p_* = 0.844) and eudaimonic well-being (F(2,284) = 218.6, *p* < 0.001, *η*^2^*_p_* = 0.606). Pairwise comparisons showed that tourists in the positive-empathy condition reported higher hedonic well-being than those in the negative-empathy condition (M_positive_ = 4.29, SD = 0.39 vs. M_negative_ = 1.73, SD = 0.64; t(284) = 34.63, *p* < 0.001) and higher eudaimonic well-being (M_positive_ = 4.19, SD = 0.47 vs. M_negative_ = 2.22, SD = 0.94; t(284) = 19.45, *p* < 0.001), supporting H1. Tourists exposed to negative empathy also scored significantly lower on both hedonic (M_negative_ = 1.73 vs. M_none_ = 4.18, SD = 0.47; t(284) = 33.01, *p* < 0.001) and eudaimonic well-being (M_negative_ = 2.21 vs. M_none_ = 3.86, SD = 0.63; t(284) = 16.16, *p* < 0.001) than the none-empathy group. Unexpectedly, although positive-empathy tourists reported higher eudaimonic well-being than the none-empathy group (M_positive_ = 4.19 vs. M_none_ = 3.87; t(284) = 3.26, *p* = 0.001), their hedonic well-being did not differ significantly (M_positive_ = 4.29 vs. M_none_ = 4.18; t(284) = 1.60, *p* > 0.05).

Restorative experience. One-way ANCOVA showed a significant effect of empathy with nature on restorative experience (F(2,284) = 699.55, *p* < 0.001, *η*^2^*_p_* = 0.831), controlling for gender and age. Pairwise comparisons revealed that tourists in the positive-empathy condition reported higher restorative experience than those in the negative-empathy condition (M_positive_ = 4.23, SD = 0.34 vs. M_negative_ = 1.96, SD = 0.66; t(284) = 32.94, *p* < 0.001). The none-empathy group also scored higher (M_none_ = 4.18, SD= 0.38 vs. M_negative_ = 1.96, SD = 0.66; t(284) = 31.71, *p* < 0.001) than the negative-empathy group, whereas restorative experience did not differ between the positive- and none-empathy conditions (M_positive_ = 4.23, SD = 0.34 vs. M_none_ = 4.18, SD = 0.38; t(284) = 0.76, *p* > 0.05).

Mediation analysis. Using PROCESS Model 4 with 5000 bootstrap samples (controlling for gender and age), with the negative-empathy condition as the reference group, restorative experience significantly mediated the effect of empathy condition on hedonic well-being, with significant indirect effects for both the no- vs. negative-empathy contrast (Effect = 1.689, Boot SE = 0.164, 95% CI [1.339, 1.983]) and the positive- vs. negative-empathy contrast (Effect = 1.730, Boot SE = 0.163, 95% CI [1.384, 2.022]), supporting H3a. Likewise, restorative experience significantly mediated the effect on eudaimonic well-being (no vs. negative: Effect = 2.216, Boot SE = 0.188, 95% CI [1.891, 2.633]; positive vs. negative: Effect = 2.269, Boot SE = 0.187, 95% CI [1.951, 2.686]), supporting H3b.

### 5.5. Experiment 3

#### 5.5.1. Purpose

Experiment 3 tests H1–H4. Beyond replicating the main and mediating effects of empathy with nature on tourist well-being and restorative experience, it examines whether wildlife anthropomorphism moderates these relationships. Using a 3 (empathy with nature: positive vs. negative vs. none) × 2 (anthropomorphism: high vs. low) between-subjects scenario experiment set in a wildlife-tourism context, we predict that when wildlife are highly anthropomorphized (relative to low-anthropomorphism), positive empathy will yield stronger restorative experience and higher well-being, and negative empathy will produce weaker restorative experience and lower well-being.

#### 5.5.2. Procedure and Materials

Experiment 3 employed a 3 × 2 between-subjects online design. For the anthropomorphism manipulation, participants first viewed a wildlife introduction panel (text plus photo, Supplementary Material S2). In the high-anthropomorphism conditions, the panel presented the animal’s name, birthday, hometown, personality, and care instructions in a warm, human-like tone; in the low-anthropomorphism conditions, it offered only scientific name, habitat, and neutral behavioural tips. Empathy with nature was manipulated exactly as in Experiment 2: three text-and-photo vignettes of equal length portrayed the animals as active/flourishing (positive empathy), distressed/lethargic (negative empathy), or calm/neutral (no empathy). At the start, participants imagined themselves on a wildlife-watching trip and were randomly assigned to one of the six combined conditions. They then completed the same 5-point scales for empathy with nature, restorative experience, hedonic well-being, and eudaimonic well-being. Anthropomorphism was checked with the item “To what extent was the red panda portrayed in a human-like way?” (1 = very low, 5 = very high), anchored by the definition “anthropomorphism refers to attributing human personality, motives, intentions, or emotions to non-human entities” (H. Zhang et al., 2024). Finally, demographics, manipulation checks, and realism check followed the same procedures used previously.

#### 5.5.3. Pilot Test

To verify that the anthropomorphism manipulation effectively altered perceived human-likeness, we conducted a pilot study. Fifty participants (37 female; M_age_ = 32.4, SD = 7.32) were recruited through Credamo and randomly assigned to view either the high- or low-anthropomorphism introduction panel. Afterward, they completed the empathy and well-being scales, rated scenario realism, and provided demographic information. Mean realism was 4.42, indicating high credibility. An independent-samples *t*-test showed that participants in the anthropomorphism condition reported significantly higher perceived anthropomorphism (M = 4.28, SD = 0.68) than those in the control condition (M = 2.44, SD = 1.00; Welch’s t(42.15) = 7.60, *p* < 0.001), confirming that the manipulation was effective and suitable for the formal experiment.

#### 5.5.4. Formal Experiment

We recruited 386 participants through Credamo; after excluding 43 who failed attention checks and 44 who had neither traveled nor engaged in any wildlife tourism in the past year, 299 valid responses remained (206 female; M_age_ = 30.9, SD = 7.33). A priori power analysis (G*Power 3.1, ANCOVA, three groups, effect size f = 0.25, α = 0.05, power = 0.80) indicated a minimum sample of 158, so the obtained sample was adequate.

Manipulation checks. Independent-samples *t*-tests showed that participants in the anthropomorphism condition perceived significantly higher anthropomorphism (M = 4.05, SD = 0.75) than those in the low-anthropomorphism condition (M = 2.36, SD = 1.06, *p* < 0.001), confirming successful anthropomorphism manipulation. Likewise, participants exposed to the positive-empathy vignette reported higher positive empathy (M = 4.29, SD = 0.37) than the no-empathy group (M = 3.87, SD = 0.71, *p* < 0.001), while those in the negative-empathy condition reported higher negative empathy (M = 4.30, SD = 0.42) than the no-empathy group (M = 3.07, SD = 0.73, *p* < 0.001), confirming successful empathy manipulation. Similarly, positive-empathy participants scored slightly higher on negative empathy than the no-empathy group (M = 3.31 vs. 3.07; Welch’s t(177.54) = 2.66, *p* = 0.008), again indicating mild mixed affect, while remaining well below the negative-empathy condition (M = 4.30).

One-way ANCOVA (controlling for gender and age) showed a significant main effect of empathy with nature on both hedonic well-being (F(2,291) = 152.43, *p* < 0.001, *η*^2^*_p_* = 0.512) and eudaimonic well-being (F(2, 291) = 45.41, *p* < 0.001, *η*^2^*_p_* = 0.238). Pairwise comparisons revealed that participants in the positive-empathy condition reported higher hedonic well-being (M = 4.31, SD = 0.29) than those in the negative-empathy condition (M = 2.66, SD = 1.11; t(291)= 15.85, *p* < 0.001) and higher eudaimonic well-being (M_positive_ = 4.16, SD = 0.49 vs. M_negative_ = 3.22, SD = 0.96; t(291) = 8.84, *p* < 0.001), supporting H1. Tourists exposed to negative empathy again scored lower on both hedonic (M_negative_ = 2.66 vs. M_none_ = 4.16, SD = 0.57; t(291) = 14.32, *p* < 0.001) and eudaimonic well-being (M_negative_ = 3.22 vs. M_none_ = 4.02, SD = 0.71; t(291) = 7.50, *p* < 0.001) than the none-empathy group. Replicating Experiment 2, the positive- and no-empathy conditions did not differ significantly in hedonic well-being (M~positive~ = 4.31 vs. M~none~ = 4.16; t(291) = 1.53, *p* = 0.128), nor in eudaimonic well-being (M~positive~ = 4.16 vs. M~none~ = 4.02; t(291) = 1.43, *p* = 0.156).

Restorative experience. One-way ANCOVA (controlling for gender and age) showed a significant main effect of empathy with nature on restorative experience (F(2, 291) = 183.79, *p* < 0.001, *η*^2^*_p_* = 0.558). Pairwise comparisons revealed that tourists in the positive-empathy condition reported greater restoration than those in the negative-empathy condition (M_positive_ = 4.31, SD = 0.30 vs. M_negative_ = 2.74, SD = 0.92; t(291) = 17.08, *p* < 0.001), and the no-empathy group also scored higher than the negative-empathy group (M_none_ = 4.20, SD = 0.54 vs. M_negative_ =2.74, SD = 0.92; t(291) = 16.02, *p*< 0.001). Once again, restorative experience did not differ between positive- and no-empathy conditions (M_positive_ = 4.31, SD = 0.30 vs. M_none_ =4.20, SD = 0.54; t(291) = 1.24, *p* = 0.214).

Mediation analysis. Using PROCESS Model 4 with 5000 bootstrap samples (controlling for gender and age), with the negative-empathy condition as the reference group, restorative experience again significantly mediated the effect of empathy condition on hedonic well-being (no vs. negative: Effect = 1.403, Boot SE = 0.114, 95% CI [1.182, 1.628]; positive vs. negative: Effect = 1.513, Boot SE = 0.108, 95% CI [1.306, 1.727]), replicating support for H3a. The same pattern held for eudaimonic well-being (no vs. negative: Effect = 1.075, Boot SE = 0.115, 95% CI [0.858, 1.307]; positive vs. negative: Effect = 1.160, Boot SE = 0.120, 95% CI [0.926, 1.402]), replicating support for H3b.

Moderation analysis. A two-way ANCOVA revealed a significant interaction between empathy with nature and anthropomorphism on hedonic well-being (F(2,291) = 4.248, *p* < 0.05, *η*^2^*_p_* = 0.028), supporting H4a. Simple-effect analyses showed that this interaction was driven primarily by the negative-empathy condition: compared with the low-anthropomorphism condition, tourists experiencing negative empathy reported significantly lower hedonic well-being when anthropomorphism was high (M_high_ = 2.44, SD = 1.04 vs. M_low_ = 2.88, SD = 1.13; MD = 0.437, SE = 0.146, *p* = 0.003). whereas the difference was not significant in the no-empathy condition (M_high_ = 4.17, SD = 0.60 vs. M_low_ = 4.14, SD = 0.55; *p* = 0.820) and, though in the expected direction, not significant in the positive-empathy condition either (M_high_ = 4.38, SD = 0.26 vs. M_low_ = 4.26, SD = 0.31; *p* = 0.396). No significant interaction was found on eudaimonic well-being (F(2, 291) = 1.474, *p* = 0.231, *η*^2^*_p_* = 0.010), supporting H4b. Additionally, a significant interaction emerged on restorative experience (F(2, 291) = 3.529, *p* = 0.031, *η*^2^*_p_* = 0.024), supporting H4c; simple effects indicated that high anthropomorphism significantly dampened restoration specifically in the negative-empathy condition (*p* = 0.005), with no effects in the other two conditions (ps > 0.49).

## 6. Discussion and Conclusions

### 6.1. Discussion

Drawing on the broaden-and-build and vicarious-experience theories, this study unpacks how empathy with nature shapes tourist well-being in wildlife-tourism settings. Study 1, through depth interviews and grounded analysis, identified two valenced facets of nature-based empathy—positive and negative—and the dual dimensions of tourist well-being (hedonic and eudaimonic). The qualitative evidence further revealed restorative experience as the pivotal mechanism linking empathy to well-being and suggested that wildlife anthropomorphism may act as a boundary condition. Three online experiments in Study 2 causally validated these pathways. Based on mainland Chinese participants in scenario-based zoo encounters, these findings offer an initial, culturally situated account of how empathy with nature shapes tourist well-being in wildlife tourism.

The findings demonstrate that empathy with nature exerts a significant influence on well-being: positive empathy yields substantially higher hedonic and eudaimonic well-being than negative empathy. Whereas earlier work has documented a positive association between empathy and life satisfaction (Cohn et al., 2009; Fredrickson et al., 2008; Shanafelt et al., 2005), it has typically treated empathy as uni-dimensional. The present research shows that, in wildlife tourism, the well-being gains arise through two intertwined routes. First, direct affective contagion operates: observing animals in a flourishing state transmits positive affect to visitors, immediately elevating hedonic well-being (Fredrickson, 2001). Second, positive empathy broadens tourists’ cognitive frame and personal resources—deepening their sense of connection to, and responsibility for, wildlife—thereby fostering meaning construction and self-realisation. One unexpected pattern merits brief note: the positive-empathy and no-empathy conditions did not differ in hedonic well-being or restorative experience, suggesting that mere nature exposure may suffice for restorative and hedonic gains, whereas positive empathy’s contribution to eudaimonic well-being was significant in Experiment 2 but not replicated in Experiment 3. We attribute this null contrast to a likely ceiling effect: imagining a visit to a zoo carries inherent positive valence, which elevates the no-empathy baseline and compresses the incremental gain afforded by positive empathy.

Taken together with the explicit positive- versus no-empathy contrasts now reported in Section 5.4.4 and Section 5.5.4, the overall pattern supports an asymmetric interpretation: negative empathy is actively harmful—it significantly depressed hedonic and eudaimonic well-being and restoration in every experiment—whereas positive empathy chiefly preserves well-being relative to negative empathy and does not reliably exceed a neutral nature encounter. One inconsistency deserves note: positive empathy significantly exceeded the no-empathy condition on eudaimonic well-being in Experiment 2 (t(284) = 3.26, *p* = 0.001) but not in Experiment 3 (t(291) = 1.43, *p* = 0.156), despite comparable samples and materials. Because the effect is small and unstable across studies, we refrain from claiming a distinctive eudaimonic benefit of positive empathy; its robust contribution is preservative—protecting well-being relative to negative empathy—rather than additive. The attenuation in Experiment 3 may reflect the wildlife-anthropomorphism introduction panel administered before the vignette, which could have elevated meaning-related responding across all conditions and compressed the contrast; this account should be tested directly in future research.

The study further reveals that tourists who experience positive empathy with wildlife report significantly stronger restorative experiences than those who experience negative empathy, and that restorative experience positively mediates the link between empathy and well-being. While earlier work has shown that simply viewing wildlife can reduce stress and yield psychological benefits (Muloin, 1998), our findings refine this insight by demonstrating that it is the emotional quality of the encounter—positive versus negative empathy—that drives restoration. Grounded in vicarious-experience theory, the mechanism operates as follows: when animals appear happy and authentic, tourists imagine themselves embedded in that carefree lifeworld, vicariously savoring ease and spontaneity, and consequently obtain physical and mental recovery. Unlike human interactions, which are often evaluative, animals offer non-judgmental presence; their companionship provides social support without threatening the self (Hughes et al., 2021; Jakoby, 2015). Positive empathy with wildlife thus functions as unconditional accompaniment, enabling tourists to discover and deploy personal strengths, confront life setbacks, and reconstruct meaning—an intrapsychic process that replenishes energy and enriches their spiritual landscape.

This study identifies wildlife anthropomorphism as a moderator of the link between empathy with nature and both restorative experience and hedonic well-being, but not eudaimonic well-being. While previous research has demonstrated that anthropomorphism can foster pro-social or conservation-oriented behaviors (Chang et al., 2020; Yue et al., 2021), it has not directly examined whether anthropomorphism alters tourists’ own restoration or well-being. Our findings reveal an asymmetric amplification pattern: anthropomorphism significantly intensified the harmful effects of negative empathy—tourists in the negative-empathy condition reported lower hedonic well-being (MD = 0.437, *p* = 0.003) and dampened restoration (MD = 0.357, *p* = 0.005) under high anthropomorphism—whereas the corresponding positive-empathy contrasts, though directionally consistent with amplification, were not significant (*p*s > 0.39). Social anthropologists argue that we instinctively project human social values onto animals and interpret their world through human referents (Milton, 2020), which helps explain why wildlife displaying “human-like” traits—family bonds, nurturing, cuteness, sociability—are perceived as more attractive (Curtin, 2005). Vicarious-experience theory offers a complementary explanation: the closer the substitute is to the self, the more intense the vicarious experience (Huang et al., 2015; McKeever, 2015). Highly anthropomorphized animals thus serve as readily identifiable substitutes, which helps explain why anthropomorphism intensifies the vicarious distress elicited by negative empathy; whether it reliably amplifies the benefits of positive empathy remains an open question for future research.

The absence of a moderating effect on eudaimonic well-being suggests that self-realization and meaning construction arise primarily through cognitive rather than affective pathways (Chu et al., 2025). Eudaimonic well-being is comparatively stable and trait-like, rooted in enduring value systems, whereas hedonic well-being is a transient state; a brief situational encounter may therefore shift emotional outcomes while leaving meaning-laden judgments largely untouched (Huta & Ryan, 2010). In wildlife tourism, eudaimonic outcomes stem from tourists’ reflections on animal welfare and their own ethical stance; these cognitions are triggered by the perceived need to care, irrespective of how “human-like” the animal appears. Because light-touch anthropomorphism conveys only surface emotions and lacks deep narrative capable of challenging or expanding visitors’ value frameworks, it does not provoke the existential reframing necessary to boost eudaimonic well-being. Moreover, meaning construction may require post-visit incubation and reflective elaboration that an immediate post-scenario measurement cannot capture; what our design detects is therefore the fast “affective channel” rather than the slower “meaning channel.” Taken together, these considerations reinforce our view that anthropomorphism operates as an affective amplifier whose influence stops at the boundary of cognitive meaning construction. Consequently, wildlife anthropomorphism influences tourist happiness mainly through emotional channels, offering new insight into how human-like portrayals of wildlife shape the quality of tourist experiences.

### 6.2. Theoretical Implications

First, this study expands the conceptual scope of empathy with nature by disaggregating it into positive and negative valences. In the broader empathy literature, negative empathy aligns with ‘empathic concern’ or ‘personal distress’ in response to others’ suffering, whereas positive empathy aligns with ‘empathic joy’—the capacity to share and be uplifted by others’ positive affect (Telle & Pfister, 2016). By foregrounding the capacity to share and be uplifted by wildlife’s positive affect, we redress the long-standing negativity bias in empathy research and demonstrate that resonating with others’ joy is equally capable of forging social bonds, and enhancing well-being. This positive lens opens new avenues for educational, organizational, and mental-health interventions that cultivate flourishing rather than merely alleviating distress. Moreover, situating these processes within wildlife tourism extends empathy scholarship from interpersonal to interspecies contexts, documenting that humans can experience genuine emotional resonance with non-human life and offering fresh psychological grounding for wildlife conservation, ecological ethics, and harmonious human–nature coexistence.

Second, we uncover the mechanism linking empathy with nature to tourist well-being. Restorative experience functions as the pivotal conduit, and we introduce “vicarious healing” as a novel theoretical lens. In today’s high-speed, hyper-competitive societies, individuals are confined to fragmented time and limited space; vicarious fulfillment has become the lowest-cost, “curve-around” strategy for realizing otherwise inaccessible aspirations. By converting unattainable natural freedom into psychologically real experiences at minimal temporal and spatial cost, vicarious satisfaction transforms empathic connection into a frequent and feasible form of tourist therapy. Embedding this mechanism within tourism studies provides a new explanatory framework for understanding how travel fosters restoration and elevates well-being.

This study proposes a new therapeutic route in tourism—nature-empathy healing—and outlines a revised paradigm in which the locus of empathic care shifts from human-other to human-nature. Moving away from the “environment-centred” geographical lens, we adopt a social-psychological framing that anchors the cure in inter-subjective empathy between tourists and wildlife. Visitors regulate their own affect and construct meaning through vicarious participation in animals’ apparent freedom, turning themselves into both patient and therapist. Our results suggest that empathy-based healing is not a unitary process but a dual-channel system. The affective channel is fast, vicarious, and amplifyable: it transmits the perceived emotional states of wildlife to tourists within seconds, and its strength can be modulated by surface-level cues such as anthropomorphism. The meaning channel is slower, reconstructive, and cue-resistant: it operates through cognitive reflection on animal welfare, ecological ethics, and one’s own life priorities, and it resists amplification by lightweight emotional cues.

We also identify a boundary condition for this empathetic cure. Wildlife anthropomorphism asymmetrically moderated these affective pathways: it significantly amplified the harm of negative empathy on hedonic well-being and restoration, did not reliably amplify the benefits of positive empathy, and did not moderate the link with eudaimonic well-being. Anthropomorphism therefore functions as an “affective amplifier” with asymmetric action: it exacerbates the damage of negative empathy along the emotion–restoration–pleasure cascade, yet lacks the depth required to reconfigure personal values or life meaning. This finding clarifies the boundary between the two paths of “emotional trigger” and “cognitive reconstruction” in empathy healing, and provides an operable theoretical coordinate for subsequent research to distinguish emotional and meaningful tourism experiences.

### 6.3. Practical Implications

Tourism sites should place tourists’ emotional experience at the core of product design and deliberately cultivate both hedonic and eudaimonic well-being. Because positive empathy with nature is associated with higher well-being than negative empathy, attractions can make “positive affect” the core product. Attractions should ensure that animals are presented in visibly good condition and that interpretive materials do not expose visitors to distressing depictions. On this foundation, operators can facilitate positive empathy through authentic animal-centred presentation and unhurried viewing conditions, while avoiding over-claimed ‘healing’ promises.

Destinations should therefore explore their restorative advantages and pursue diversified, differentiated growth paths. Our findings show that empathy with nature enhances restorative experience and, in turn, tourist well-being, so parks and wildlife sites must actively market their “nature-empathy value.” Product design must recognize that the new paradigm transfers the healing role from professional therapists to tourists themselves, lowering intervention thresholds and turning empathy with nature into a low-cost, accessible self-help resource. Anchoring offerings on this low-cost, high-value promise gives destinations a fresh, differentiated, and sustainable competitive narrative.

Destinations should therefore prioritise a “low-cognition, high-affect” anthropomorphism tactic that maximises healing pay-offs from positive empathy while demanding the least cognitive effort. Our results show that anthropomorphism amplifies the empathy → restoration → pleasure chain but does not penetrate the cognitive-value layer required for eudaimonic well-being; shallow semantics and time pressure keep it locked at the affect-boost stage. If managers want word-of-mouth for accessible affective enhancement, they should abandon traditional didactic stories and value-laden interpretation and instead design lightweight, easy-to-process emotional cues—cute names, playful fonts, emojis, one-second eye contact in photos. Interventions aimed at meaning construction could be piloted in practice, but the present data neither test nor support marketing them as proven routes to eudaimonic well-being.

It is worth noting that the moderating effects of anthropomorphism were small, implying that the benefits of anthropomorphism are incremental rather than substantial. Importantly, given the asymmetric moderation pattern observed in Experiment 3, destinations should apply anthropomorphism selectively as a risk-management measure: highly anthropomorphized portrayals are best suited to settings where animal welfare is visibly good and animals appear content, because anthropomorphizing animals in distress can amplify the harmful effects of negative empathy on tourists’ well-being. Anthropomorphism should therefore not be treated as a universally beneficial tactic, and attractions with evident animal-welfare problems should remedy the animals’ conditions before employing human-like portrayals. Overall, destinations should thus regard anthropomorphism as a bounded affective tactic whose value lies in careful, context-appropriate application.

### 6.4. Limitations and Future Directions

First of all, this study has some limitations in the research sample. Based on the feasibility of wildlife ethics and field experiments, this study mainly uses online situational experiments for data collection. Online data collection methods make it difficult for some younger child tourists and older silver-haired tourists to participate, and they also belong to the research object of this paper in wildlife tourism. Future work should combine on-site and online approaches to capture children’s and seniors’ authentic wildlife experiences.

Second, the study is culturally bounded. Because all respondents were mainland Chinese, the findings offer a distinctive Chinese lens but may not transfer to Western settings where tourist traits and cultural narratives differ. Chinese cultural traditions emphasizing harmony with nature (tian ren he yi) and the relational self may facilitate stronger empathy-with-nature responses than in Western individualistic contexts (Joshanloo, 2014). Moreover, the cognitive pathways to eudaimonia may be culturally shaped, meaning that the null effect of anthropomorphism on eudaimonic well-being may not replicate in cultures where anthropomorphic storytelling is less normative. Comparative studies across cultures are needed to test the generalisability of the empathy–restoration–well-being chain.

Third, although our title invokes empathy as a “double-edged sword,” we focused on well-being gains and did not measure the potential costs of empathy, such as emotional exhaustion or compassion fatigue (Andreychik, 2019; O’Connor et al., 2007). Whether sustained empathy with wildlife carries such costs remains an open question.

Fourth, the experimental design of this study has some limitations. The vignettes used to induce empathy are text-based and supplemented with static images; while realism checks confirmed credibility, the emotional responses elicited by reading about a hypothetical zoo visit may not fully correspond to the embodied, multisensory empathy arising in real wildlife encounters. The pattern of results may differ in field settings where tourists encounter animals in their natural habitats rather than in zoological settings. We therefore frame these experiments as an early-stage causal test of a theoretically derived model, and explicitly call for field-based replication using experience-sampling or observational methods. Similarly, the anthropomorphism manipulation was deliberately lightweight: short text labels and single photos. Such minimal semantic cues may be insufficient to activate the default-mode network or to prompt tourists to integrate their own life narratives with the animal’s story, thereby leaving eudaimonic well-being untouched. In addition, the experiments were cross-sectional, with well-being measured immediately after a single exposure; the durability of the empathy–restoration–well-being effects is therefore unknown. Longitudinal designs with delayed and repeated measurements are needed to distinguish transient mood shifts from lasting well-being change.

Fifth, all outcome variables were self-reported in a single session, raising the possibility of common method bias. We note, however, that the core independent variable was experimentally manipulated rather than self-reported, which structurally limits method variance for the central causal tests; that procedural remedies (anonymity, filler items between the scenario and the outcomes, established scales) were applied; and that the positive- and negative-empathy scales—the constructs most vulnerable to undifferentiated responding—were uncorrelated or negatively correlated across experiments (r = −0.74, −0.14, and −0.06, ns, in Experiments 1–3). These patterns make a common-method account unlikely, although single-session self-report measurement remains a limitation that future multi-method designs could overcome.

In the future research, it is necessary to carry out “emotion-cognition” diversified design of wildlife anthropomorphism to test what kind of intensity and form of wildlife anthropomorphism can cross the threshold of hedonic–eudaimonic, so as to clarify the psychological boundary and neural mechanism of wildlife anthropomorphism to promote or inhibit the eudaimonic well-being. In addition, future research is needed to extend these findings from emotional experiences to behavioral outcomes, thereby broadening the practical value of the research.

## Figures and Tables

**Table 1:** Research Framework.

	Experiment 1	Experiment 2	Experiment 3
Objective	To test the main effect (H1)	To test the main effect and the mediating effect (H1-H3)	To test the main effect, mediating effect and moderating effect (H1–H4)
Independent variable	Empathy with nature	Empathy with nature	Empathy with nature
Dependent variable	Hedonic well-being, eudaimonic well-being	Hedonic well-being, eudaimonic well-being	Hedonic well-being, eudaimonic well-being
Mediating variable		Restorative experience	Restorative experience
Moderating variable			Wildlife anthropomorphism

**Table 2:** Reports descriptive statistics and reliabilities for all measures.

Experiment	Scale	M	SD	α
Exp 1 (N = 146)	Positive empathy	3.77	0.65	0.935
	Negative empathy	3.92	0.68	0.955
	Hedonic well-being	3.24	1.25	0.964
	Eudaimonic well-being	3.50	1.07	0.933
Exp 2 (N = 289)	Positive empathy	3.62	0.74	0.939
	Negative empathy	3.47	0.90	0.951
	Hedonic well-being	3.39	1.29	0.945
	Eudaimonic well-being	3.42	1.12	0.968
	Restorative experience	3.45	1.17	0.943
Exp 3 (N = 299)	Positive empathy	3.81	0.68	0.907
	Negative empathy	3.56	0.78	0.935
	Hedonic well-being	3.71	1.05	0.951
	Eudaimonic well-being	3.80	0.85	0.898
	Restorative experience	3.75	0.96	0.890

## Data Availability

The data presented in this study are available upon request from the corresponding author due to privacy restrictions.

## Supplementary Materials

The following supporting information can be downloaded at: https://www.mdpi.com/article/10.3390/bs16101935/s1, Supplementary Material S1: Interview Outline; Supplementary Material S2: Experimental Materials; Supplementary Material S3: Questionnaire Scales.

## References

[B1-behavsci-16-01935] Andreychik M. R. (2019). Feeling your joy helps me to bear feeling your pain: Examining associations between empathy for others’ positive versus negative emotions and burnout. Personality and Individual Differences.

[B2-behavsci-16-01935] Andreychik M. R., Migliaccio N. (2015). Empathizing with others’ pain versus empathizing with others’ joy: Examining the separability of positive and negative empathy and their relation to different types of social behaviors and social emotions. Basic and Applied Social Psychology.

[B3-behavsci-16-01935] Ballantyne R., Packer J., Hughes K., Dierking L. (2007). Conservation learning in wildlife tourism settings: Lessons from research in zoos and aquariums. Environmental Education Research.

[B4-behavsci-16-01935] Batson C. D. (2011). Altruism in humans.

[B5-behavsci-16-01935] Batson C. D., Lishner D. A., Stocks E. L. (2015). The empathy-altruism hypothesis. The oxford handbook of prosocial behavior.

[B6-behavsci-16-01935] Bell S. L., Westley M., Lovell R., Wheeler B. W. (2018). Everyday green space and experienced well-being: The significance of wildlife encounters. Landscape Research.

[B7-behavsci-16-01935] Chang C. C., Cheng G. J. Y., Nghiem T. P. L., Song X. P., Oh R. R. Y., Richards D. R., Carrasco L. R. (2020). Social media, nature, and life satisfaction: Global evidence of the biophilia hypothesis. Scientific Reports.

[B8-behavsci-16-01935] Charles F., Hamid N. A. (2022). Economic and social benefits of wildlife tourism in Malaysia; A review. Journal of Contemporary Social Science and Educational Studies.

[B9-behavsci-16-01935] Chen J., Wang B. (2022). Mobilising therapeutic landscapes: Lifestyle migration of the Houniao and the spatio-temporal encounters with nature. Geoforum.

[B10-behavsci-16-01935] Chen Y., Lin Z., Filieri R., Liu R. (2021). Subjective well-being, mobile social media and the enjoyment of tourism experience: A broaden-and-build perspective. Asia Pacific Journal of Tourism Research.

[B11-behavsci-16-01935] Chu H., Ji C., Tian F., Prentice C. (2025). Engaging with ethnic minority tourism for tourist hedonic and eudaimonic well-being. International Journal of Tourism Research.

[B12-behavsci-16-01935] Clayton S., Fraser J., Saunders C. D. (2009). Zoo experiences: Conversations, connections, and concern for animals. Zoo Biology: Published in affiliation with the American Zoo and Aquarium Association.

[B13-behavsci-16-01935] Cohn M. A., Fredrickson B. L., Brown S. L., Mikels J. A., Conway A. M. (2009). Happiness unpacked: Positive emotions increase life satisfaction by building resilience. Emotion.

[B14-behavsci-16-01935] Coolman A. A., Niedbalski A., Powell D. M., Kozlowski C. P., Franklin A. D., Deem S. L. (2020). Changes in human health parameters associated with an immersive exhibit experience at a zoological institution. PLoS ONE.

[B15-behavsci-16-01935] Corral-Verdugo V. (2012). The positive psychology of sustainability. Environment, Development and Sustainability.

[B16-behavsci-16-01935] Cosmides L., Tooby J. (2000). Evolutionary psychology and the emotions. Handbook of Emotions.

[B17-behavsci-16-01935] Curtin S. (2005). Nature, wild animals and tourism: An experiential view. Journal of Ecotourism.

[B18-behavsci-16-01935] Curtin S. (2009). Wildlife tourism: The intangible, psychological benefits of human–wildlife encounters. Current Issues in Tourism.

[B19-behavsci-16-01935] Curtin S., Kragh G. (2014). Wildlife tourism: Reconnecting people with nature. Human Dimensions of Wildlife.

[B20-behavsci-16-01935] Di Fabio A., Kenny M. E. (2021). Connectedness to nature, personality traits and empathy from a sustainability perspective. Current Psychology.

[B21-behavsci-16-01935] Eisenberg N., Fabes R. A. (1990). Empathy: Conceptualization, measurement, and relation to prosocial behavior. Motivation and Emotion.

[B22-behavsci-16-01935] Epley N., Waytz A., Akalis S., Cacioppo J. T. (2008). When we need a human: Motivational determinants of anthropomorphism. Social Cognition.

[B23-behavsci-16-01935] Farkić J., May C., Pomfret G., Sand M., Shahvali M. (2025). Restoring ‘love of life’ through outdoor adventures: A biophilic approach. International Journal of Tourism Research.

[B24-behavsci-16-01935] Fennell D. A. (2024). Empathy in animal-based tourism contrasting constructed care and care ethics at a captive wildlife venue. Tourism Recreation Research.

[B25-behavsci-16-01935] Fennell D. A., Coose S., Moorhouse T. P. (2023). An animal welfare syllabus for wildlife tourism. Journal of Sustainable Tourism.

[B26-behavsci-16-01935] Fido D., Richardson M. (2019). Empathy mediates the relationship between nature connectedness and both callous and uncaring traits. Ecopsychology.

[B27-behavsci-16-01935] Fredrickson B. L. (2001). The role of positive emotions in positive psychology: The broaden-and-build theory of positive emotions. American Psychologist.

[B28-behavsci-16-01935] Fredrickson B. L., Branigan C. (2005). Positive emotions broaden the scope of attention and thought-action repertoires. Cognition & Emotion.

[B29-behavsci-16-01935] Fredrickson B. L., Cohn M. A., Coffey K. A., Pek J., Finkel S. M. (2008). Open hearts build lives: Positive emotions, induced through loving-kindness meditation, build consequential personal resources. Journal of Personality and Social Psychology.

[B30-behavsci-16-01935] Freud S. (1989). The ego and the id (1923). Tacd Journal.

[B31-behavsci-16-01935] Freud S. (2015). Civilization and its discontents.

[B32-behavsci-16-01935] Garland E. L., Fredrickson B. L. (2019). Positive psychological states in the arc from mindfulness to self-transcendence: Extensions of the mindfulness-to-meaning theory and applications to addiction and chronic pain treatment. Current Opinion in Psychology.

[B33-behavsci-16-01935] Glaser B., Strauss A. (2017). Discovery of grounded theory: Strategies for qualitative research.

[B34-behavsci-16-01935] Gray H. M., Gray K., Wegner D. M. (2007). Dimensions of mind perception. Science.

[B35-behavsci-16-01935] Graziano W. G., Habashi M. M., Sheese B. E., Tobin R. M. (2007). Agreeableness, empathy, and helping: A person× situation perspective. Journal of Personality and Social Psychology.

[B36-behavsci-16-01935] Grotto G., Buja A. (2026). Quantitative and qualitative analysis of the effects of human-animal interactions on health and well-being of older adults: An umbrella review. BMC Geriatrics.

[B37-behavsci-16-01935] Guthrie S. E. (1995). Faces in the clouds: A new theory of religion.

[B38-behavsci-16-01935] Hall C. S. (2016). A primer of Freudian psychology.

[B40-behavsci-16-01935] Han B., Wang L., Li X. (2020). To collaborate or serve? Effects of anthropomorphized brand roles and implicit theories on consumer responses. Cornell Hospitality Quarterly.

[B39-behavsci-16-01935] Han B. C. (2015). The burnout society.

[B41-behavsci-16-01935] Hehir C., Scarles C., Wyles K. J., Kantenbacher J. (2023). Last chance for wildlife: Making tourism count for conservation. Journal of Sustainable Tourism.

[B42-behavsci-16-01935] Higginbottom K., Higginbottom K. (2004). Wildlife tourism: An introduction. Wildlife tourism: Impacts, management and planning.

[B43-behavsci-16-01935] Hinds J., Sparks P. (2008). Engaging with the natural environment: The role of affective connection and identity. Journal of Environmental Psychology.

[B44-behavsci-16-01935] Huang J. L., Ryan A. M., Mujtaba B. G. (2015). Vicarious experience of justice: When unfair treatment of one’s colleague matters. Personnel Review.

[B45-behavsci-16-01935] Hughes A. M., Braun L., Putnam A., Martinez D., Fine A. (2021). Advancing human–animal interaction to counter social isolation and loneliness in the time of COVID-19: A model for an interdisciplinary public health consortium. Animals.

[B46-behavsci-16-01935] Huta V., Ryan R. M. (2010). Pursuing pleasure or virtue: The differential and overlapping well-being benefits of hedonic and eudaimonic motives. Journal of Happiness Studies.

[B47-behavsci-16-01935] Jakoby N. R. (2015). The self and significant others: Toward a sociology of loss. Illness, Crisis & Loss.

[B48-behavsci-16-01935] Jia R., Xu H., Huang C., Ma Y., Wei Z. (2026). How nature adventure tourism heals employees’ unhappiness: A logotherapy approach. Tourism Management.

[B49-behavsci-16-01935] Jing K., Qi M., Mei Y., Chen L. (2022). The impact of empathy with nature on green purchase behavior: An ERP study. Neuroscience Letters.

[B50-behavsci-16-01935] Joshanloo M. (2014). Eastern conceptualizations of happiness: Fundamental differences with western views. Journal of Happiness Studies.

[B51-behavsci-16-01935] Kandaurova M., Lee S. H. M. (2019). The effects of virtual reality (VR) on charitable giving: The role of empathy, guilt, responsibility, and social exclusion. Journal of Business Research.

[B52-behavsci-16-01935] Kaplan R., Kaplan S., Brown T. (1989). Environmental preference: A comparison of four domains of predictors. Environment and Behavior.

[B53-behavsci-16-01935] Kline C. S., Hoarau-Heemstra H., Cavaliere C. T. (2023). Wildlife equity theory for multispecies tourism justice. Journal of Travel Research.

[B54-behavsci-16-01935] Korpela K. M., Ylén M., Tyrväinen L., Silvennoinen H. (2008). Determinants of restorative experiences in everyday favorite places. Health & Place.

[B55-behavsci-16-01935] Larionow P. (2025). The dark and light sides of empathy: The clinical relevance of the assessment of cognitive and affective empathy across negative and positive emotions. European Journal of Investigation in Health, Psychology and Education.

[B56-behavsci-16-01935] Li Y., Zhou X., Jiang X., Fan F., Song B. (2024). How service robots’ human-like appearance impacts consumer trust: A study across diverse cultures and service settings. International Journal of Contemporary Hospitality Management.

[B57-behavsci-16-01935] Liao H. (2007). Do it right this time: The role of employee service recovery performance in customer-perceived justice and customer loyalty after service failures. Journal of Applied Psychology.

[B58-behavsci-16-01935] Liu Y., Ning S., Zhang M., Font X., Zeng H. (2024). Can anthropomorphic interpretation cues motivate tourists to have civilized behavioral intentions? The roles of meaningful experience and narrative. Tourism Management.

[B59-behavsci-16-01935] Lundin E., Soulard J., Stewart W. (2023). A broaden-and-build theoretical perspective on dark tourism visitors’ transformative emotional journeys. Leisure Sciences.

[B60-behavsci-16-01935] Ma J., Tu H., Zhou X., Niu W. (2023). Can brand anthropomorphism trigger emotional brand attachment?. The Service Industries Journal.

[B61-behavsci-16-01935] McIntosh D., Wright P. A. (2017). Emotional processing as an important part of the wildlife viewing experience. Journal of Outdoor Recreation and Tourism.

[B62-behavsci-16-01935] McKeever R. (2015). Vicarious experience: Experimentally testing the effects of empathy for media characters with severe depression and the intervening role of perceived similarity. Health Communication.

[B63-behavsci-16-01935] Mehrabian A. (2000). Beyond IQ: Broad-based measurement of individual success potential or” emotional intelligence”. Genetic, Social, and General Psychology Monographs.

[B64-behavsci-16-01935] Methorst J., Arbieu U., Bonn A., Boehning-Gaese K., Mueller T. (2020). Non-material contributions of wildlife to human well-being: A systematic review. Environmental Research Letters.

[B65-behavsci-16-01935] Milton K. (2020). Anthropomorphism or egomorphism? The perception of non-human persons by human ones. Animals in person.

[B66-behavsci-16-01935] Muloin S. (1998). Wildlife tourism: The psychological benefits of whale watching. Pacific Tourism Review.

[B67-behavsci-16-01935] O’Connor L. E., Berry J. W., Lewis T., Mulherin K., Crisostomo P. S. (2007). Empathy and depression: The moral system on overdrive. Empathy in Mental Illness.

[B68-behavsci-16-01935] Odendaal J. S., Lehmann S. M. C. (2000). The role of phenylethylamine during positive human-dog interaction. Acta Veterinaria Brno.

[B69-behavsci-16-01935] Ouerghemmi C., Ertz M., Bouslama N., Tandon U. (2023). The impact of virtual reality (VR) tour experience on tourists’ intention to visit. Information.

[B70-behavsci-16-01935] Park S., Ahn D. (2022). Seeking pleasure or meaning? The different impacts of hedonic and eudaimonic tourism happiness on tourists’ life satisfaction. International Journal of Environmental Research and Public Health.

[B71-behavsci-16-01935] Perry A., Shamay-Tsoory S. (2013). Understanding emotional and cognitive empathy: A neuropsychological perspective. Understanding other minds: Perspectives from developmental social neuroscience.

[B72-behavsci-16-01935] Perry D. J., Crawford S. L., Averka J. J., Mackin J. M., Granger D. A., Smelson D. A. (2025). Wildlife and wellbeing: Wildlife immersion experiences in veterans with PTSD. Human-Animal Interactions.

[B73-behavsci-16-01935] Preylo B. D., Arikawa H. (2008). Comparison of vegetarians and non-vegetarians on pet attitude and empathy. Anthrozoös.

[B74-behavsci-16-01935] Rehman M. A., Oikarinen E. L., Söderlund M. (2025). Understanding the sources of happiness in an extended tourism context: A longitudinal interpretative phenomenological analysis. International Journal of Tourism Research.

[B75-behavsci-16-01935] Rizzolo J. B. (2023). Wildlife tourism and consumption. Journal of Sustainable Tourism.

[B76-behavsci-16-01935] Root-Bernstein M., Douglas L., Smith A., Verissimo D. (2013). Anthropomorphized species as tools for conservation: Utility beyond prosocial, intelligent and suffering species. Biodiversity and Conservation.

[B77-behavsci-16-01935] Ryan R. M., Deci E. L. (2001). On happiness and human potentials: A review of research on hedonic and eudaimonic well-being. Annual Review of Psychology.

[B78-behavsci-16-01935] Sassenrath C., Diefenbacher S., Siegel A., Keller J. (2016). A person-oriented approach to hand hygiene behaviour: Emotional empathy fosters hand hygiene practice. Psychology & Health.

[B79-behavsci-16-01935] Schultz P. W. (2000). New environmental theories: Empathizing with nature: The effects of perspective taking on concern for environmental issues. Journal of Social Issues.

[B80-behavsci-16-01935] Shackley M. (1996). Wildlife tourism.

[B81-behavsci-16-01935] Shanafelt T. D., West C., Zhao X., Novotny P., Kolars J., Habermann T., Sloan J. (2005). Relationship between increased personal well-being and enhanced empathy among: Internal medicine residents. Journal of General Internal Medicine.

[B82-behavsci-16-01935] Shen Q., Wu Y., Li X., Lu J. (2023). College students’ aesthetic affection and prosocial behaviour: The role of empathy. Journal of Psychology in Africa.

[B83-behavsci-16-01935] Sirgy M. J., Uysal M. (2016). Developing a eudaimonia research agenda in travel and tourism. Handbook of eudaimonic well-being.

[B84-behavsci-16-01935] Smith M. K., Diekmann A. (2017). Tourism and wellbeing. Annals of Tourism Research.

[B85-behavsci-16-01935] Soyoren S. A., Aktas B. E. (2024). Personal belief in a just world moderates the link between induced empathy and willingness to volunteerism among non-volunteers, but not volunteers. VOLUNTAS: International Journal of Voluntary and Nonprofit Organizations.

[B86-behavsci-16-01935] Sthapit E., Ji C., Dayour F., Badu-Baiden F. (2024). Memorable wildlife tourism experience: Evidence from the mole national park. Journal of Destination Marketing & Management.

[B87-behavsci-16-01935] Strauss A., Corbin J. (1990). Basics of qualitative research.

[B88-behavsci-16-01935] Su L., Yang X., Huang Y. (2022). How do tourism goal disclosure motivations drive Chinese tourists’ goal-directed behaviors? The influences of feedback valence, affective rumination, and emotional engagement. Tourism Management.

[B89-behavsci-16-01935] Sung J. Y., Han J. S. (2023). Exploring the role of empathy as a dual mediator in the relationship between human–pet attachment and quality of life: A survey study among adult dog owners. Animals.

[B90-behavsci-16-01935] Tam K. P. (2013). Dispositional empathy with nature. Journal of Environmental Psychology.

[B91-behavsci-16-01935] Teh C., Liu L. L., Phang C. W. (2026). Eliciting pro-environmental behavior with immersive technology: The roles of awe, empathy with nature, and perceived consumer effectiveness. Information & Management.

[B92-behavsci-16-01935] Telle N. T., Pfister H. R. (2016). Positive empathy and prosocial behavior: A neglected link. Emotion Review.

[B93-behavsci-16-01935] Valença T., Resende B., Savalli C. (2021). Human–wildlife interactions with different species in a Brazilian park: A naturalistic approach. Anthrozoös.

[B94-behavsci-16-01935] Van den Born R. J., Arts B., Admiraal J., Beringer A., Knights P., Molinario E., Horvat K. P., Porras-Gomez C., Smrekar A., Soethe N., Vivero-Pol J., Ganzevoort W., Bonaiuto M., Knippenberg L., De Groot W. (2018). The missing pillar: Eudemonic values in the justification of nature conservation. Journal of Environmental Planning and Management.

[B95-behavsci-16-01935] Vidon E. S., Rickly J. M., Knudsen D. C. (2018). Wilderness state of mind: Expanding authenticity. Annals of Tourism Research.

[B96-behavsci-16-01935] Wang C., Lu C. (2025). The effect of awe in wildlife tourists on animal-friendly behavioural intentions: A serial mediation model of connectedness to nature and empathy. Asia Pacific Journal of Tourism Research.

[B97-behavsci-16-01935] Wang K., Huang S., Chen Z., Liu Y. (2026). The impact of empathy with nature on tourists’ responsible behavioral intentions in natural heritage tourism: The moderating roles of escapism and awe. Frontiers in Psychology.

[B98-behavsci-16-01935] Wang L., Sheng G., She S., Xu J. (2023). Impact of empathy with nature on pro-environmental behaviour. International Journal of Consumer Studies.

[B99-behavsci-16-01935] Wang X., Ming M., Zhang Y. (2020). Are “people” or “animals” more attractive? Anthropomorphic images in green-product advertising. Journal of Cleaner Production.

[B100-behavsci-16-01935] Waterman A. S. (1993). Two conceptions of happiness: Contrasts of personal expressiveness (eudaimonia) and hedonic enjoyment. Journal of Personality and Social Psychology.

[B101-behavsci-16-01935] Westbury H. R., Neumann D. L. (2008). Empathy-related responses to moving film stimuli depicting human and non-human animal targets in negative circumstances. Biological Psychology.

[B102-behavsci-16-01935] White M. E., Hamlin I., Butler C. W., Richardson M. (2023). The joy of birds: The effect of rating for joy or counting garden bird species on wellbeing, anxiety, and nature connection. Urban Ecosystems.

[B103-behavsci-16-01935] Wu X., Lu X. J., Zhang L. B., Huo S. R., Kong Y. Z., Hu L. (2025). Exploring empathic responses to others’ positive and negative experiences: Insights from empathy subprocesses. Neuroscience Bulletin.

[B104-behavsci-16-01935] Xiong W., Luo X., Leung X. Y., Huang M. (2025). Unraveling meaning in life from tourism: An iconology approach on cancer patients. Tourism Management Perspectives.

[B106-behavsci-16-01935] Yerbury R., Weiler B. (2020). From human wellbeing to an ecocentric perspective: How nature-connectedness can extend the benefits of marine wildlife experiences. Anthrozoös.

[B105-behavsci-16-01935] Yerbury R. M., Boyd W. E. (2019). Dolphins and human flourishing: A novel application of the PERMA model. Ecopsychology.

[B107-behavsci-16-01935] Yu J., Smale B., Xiao H. (2021). Examining the change in wellbeing following a holiday. Tourism Management.

[B108-behavsci-16-01935] Yue D., Tong Z., Tian J., Li Y., Zhang L., Sun Y. (2021). Anthropomorphic strategies promote wildlife conservation through empathy: The moderation role of the public epidemic situation. International Journal of Environmental Research and Public Health.

[B109-behavsci-16-01935] Zhang H., Qiu S. C., Wang X., Yuan X. (2024). Robots or humans: Who is more effective in promoting hospitality services?. International Journal of Hospitality Management.

[B110-behavsci-16-01935] Zhang Y., Lee T. J., Xiong Y. (2022). The contribution of animal-based cultural ecosystem services to tourist well-being at cultural heritage sites. Journal of Travel Research.

