# Supplementary material for: Is Empathy a “Double-Edged Sword”? How Empathy with Nature Shapes Tourist Well-Being in Wildlife Tourism
Source: Behav Sci (Basel). 2026 Oct 17;16(10):1935. doi: 10.3390/bs16101935 (PMC13701337; doi:10.3390/bs16101935)
Supplement: Supplementary file 1 [file behavsci-16-01935-s001.zip › behavsci-4489887-supplementary.pdf]

Supplementary Material S1: Interview Outline

Thank you for agreeing to participate in this study. The purpose of the interview is to understand your experiences and feelings regarding wildlife tourism undertaken within the past twelve months. The interview is anonymous, contains no sensitive personal questions, and will last approximately 30–60 minutes. Please answer on the basis of your actual experiences and perceptions. Your participation is greatly appreciated.

1. Please recall and describe the general circumstances of your most recent wildlife tourism experience (time, destination, travel companions, weather conditions, etc.).
2. Please recall and describe your feelings when you encountered wildlife at close quarters, including your immediate reactions and any bodily sensations.
3. Did the wildlife tourism experience generate a sense of well-being? What exactly happened? Please describe the situation in detail.
4. In what specific ways did this experience enhance your sense of well-being? Could you elaborate on the feelings or thoughts involved?
5. Did the resulting increase in well-being influence your subsequent study, work, or daily life?
6. Do you think the sense of well-being brought by this wildlife tourism experience is important for your choice of a travel destination?
7. How long did the sense of well-being brought by that trip last? Was there any relationship between the duration of well-being and the wildlife experience?

Thank you very much for your support and assistance. Best wishes!

Supplementary Material S2: Experimental Materials

1. Materials for Experiment 1

Contextual activation	On an early weekend morning, you arrive at the nearby zoo in high spirits, eager to explore its wondrous world.<br>At the crowded entrance, you wait in line to buy a ticket, anticipating the adventure about to unfold. Finally, you receive your pass to this realm of fantasy—the colorful ticket ushers you into this paradise.<br>Once inside, you follow the flow of visitors forward, and before your eyes, various animals appear, each displaying its unique demeanor within its own home.
Positive natural empathy situation (225	On the lake surface, the swans gracefully swam. From time to time, they lowered their heads and used their beaks to comb their feathers, then continued moving forward. Some swans would suddenly spread their wings and gently slap the water, splashing a series of water droplets, looking both free and happy.<br>On the Monkey Hill, the monkeys were busy. They moved among the branches, some

words)	chasing and playing, some picking fruits to eat. The little monkeys tightly held onto their mothers, looking around curiously. Occasionally, a monkey would stop and make a funny face at the tourists.<br>In the Panda Pavilion, the pandas were enjoying their lunch. They sat on the ground, using their front paws to skillfully peel the bamboo leaves and eating them in large mouthfuls. Some pandas, when they were full, would roll on the ground, revealing their round bellies, looking particularly content.<br>In the Giraffe Area, these tall animals were stretching their necks to pick leaves from the trees to eat. Their movements were elegant and slow, and from time to time, they would lower their heads, using their big eyes to curiously look around. Throughout the zoo, the animals seemed very at ease. They ate or played, rested or explored, each one showing its unique personality and vitality.<br>Then, you visited other exhibits and experienced the entire zoo. After that, you left the zoo.
Negative natural empathy situation (188 words)	On the lake surface, the swans were floating quietly, their feathers losing their former luster. They no longer swam gracefully but appeared somewhat heavy, occasionally raising their heads to look at the sky, seemingly reminiscing about the days of free flight.<br>On the Monkey Hill, the monkeys were no longer as lively as usual. Some of them sat quietly in the corner, their eyes revealing a deep melancholy. The little monkeys clung tightly to their mothers, no longer exploring around curiously as before. Occasionally, a monkey would emit a low sound, seemingly telling about their loneliness.<br>In the Panda Pavilion, the pandas lay quietly in the corner, losing interest in the bamboo in front of them. Their eyes revealed a deep melancholy, as if thinking about something. Some pandas just sat quietly, motionless, seemingly expressing their unhappiness in silence.<br>In the Giraffe Area, the giraffes stood quietly, their eyes lacking the former curiosity and vitality. They just looked towards the distance, seemingly remembering the vast grassland and the days of free running.<br>Then, you visited other exhibits and experienced the entire zoo. After that, you left the zoo.

2. Materials for Experiment 2

Contextual activation	On an early weekend morning, you arrive at the nearby zoo in high spirits, eager to explore its wondrous world.<br>At the crowded entrance, you wait in line to buy a ticket, anticipating the adventure about to unfold. Finally, you receive your pass to this realm of fantasy—the colorful ticket ushers you into this paradise.<br>Once inside, you follow the flow of visitors forward, and before your eyes, various animals appear, each displaying its unique demeanor within its own home.

<table>
<tr>
<td rowspan="2">Positive natural empathy situation (225 words)</td>
<td colspan="2">On the lake surface, the swans gracefully swam. From time to time, they lowered their heads and used their beaks to comb their feathers, then continued moving forward. Some swans would suddenly spread their wings and gently slap the water, splashing a series of water droplets, looking both free and happy.
On the Monkey Hill, the monkeys were busy. They moved among the branches, some chasing and playing, some picking fruits to eat. The little monkeys tightly held onto their mothers, looking around curiously. Occasionally, a monkey would stop and make a funny face at the tourists.
In the Panda Pavilion, the pandas were enjoying their lunch. They sat on the ground, using their front paws to skillfully peel the bamboo leaves and eating them in large mouthfuls. Some pandas, when they were full, would roll on the ground, revealing their round bellies, looking particularly content.
In the Giraffe Area, these tall animals were stretching their necks to pick leaves from the trees to eat. Their movements were elegant and slow, and from time to time, they would lower their heads, using their big eyes to curiously look around. Throughout the zoo, the animals seemed very at ease. They ate or played, rested or explored, each one showing its unique personality and vitality.
Then, you visited other exhibits and experienced the entire zoo. After that, you left the zoo.</td>
</tr>
<tr>
<td>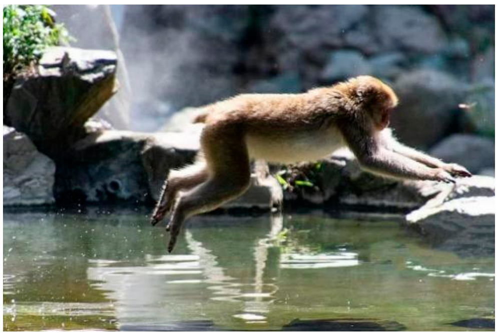</td>
<td>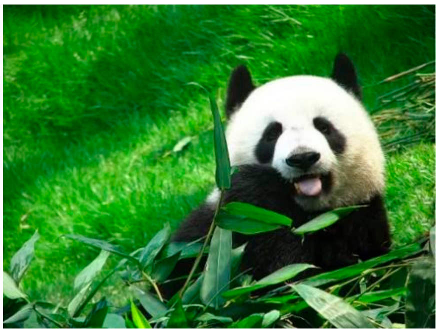</td>
</tr>
<tr>
<td>Negative natural empathy situation (188 words)</td>
<td colspan="2">On the lake surface, the swans were floating quietly, their feathers losing their former luster. They no longer swam gracefully but appeared somewhat heavy, occasionally raising their heads to look at the sky, seemingly reminiscing about the days of free flight.
On the Monkey Hill, the monkeys were no longer as lively as usual. Some of them sat quietly in the corner, their eyes revealing a deep melancholy. The little monkeys clung tightly to their mothers, no longer exploring around curiously as before. Occasionally, a monkey would emit a low sound, seemingly telling about their loneliness.
In the Panda Pavilion, the pandas lay quietly in the corner, losing interest in the bamboo in front of them. Their eyes revealed a deep melancholy, as if thinking about something. Some pandas just sat quietly, motionless, seemingly expressing their unhappiness in silence.
In the Giraffe Area, the giraffes stood quietly, their eyes lacking the former curiosity and vitality. They just looked towards the distance, seemingly remembering the vast grassland and the days of free running.
Then, you visited other exhibits and experienced the entire zoo. After that, you left the zoo.</td>
</tr>
</table>

<table>
<tr><td></td><td>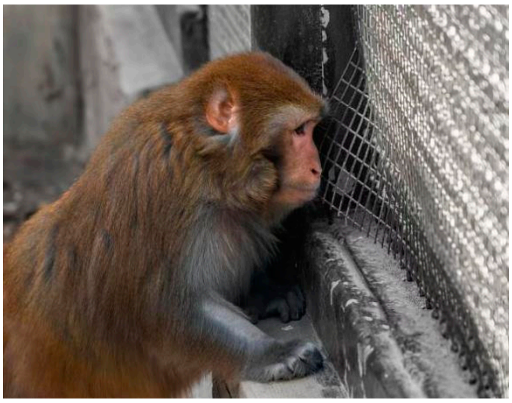</td><td>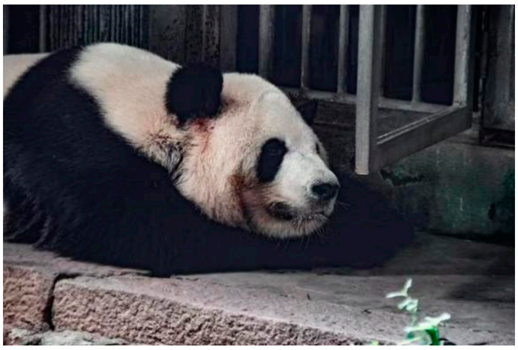</td></tr>
<tr><td rowspan="2">No natural empathy situation (195 words)</td><td colspan="2">On the lake surface, the swans were gliding gently on the water, occasionally using their beaks to touch the water and maintaining their balance. They would sometimes look up at the sky, then lower their heads and continue swimming, moving slowly and rhythmically.
On the Monkey Hill, the monkeys were moving quietly among the branches. Some were concentrating on picking leaves, while others were leisurely grooming their fur. The little monkeys were closely clinging to their mothers, occasionally looking around curiously but without much movement.
In the Panda Pavilion, the pandas were sitting on the ground, using their front paws to peel off bamboo leaves and eating attentively. After eating, some pandas would lie down on the ground, occasionally changing positions, and lying quietly, occasionally gently patting their bellies with their paws.
In the Giraffe Area, the giraffes were stretching their necks to pick fresh leaves from the trees. They would occasionally lower their heads to observe their surroundings, then continue eating grass quietly. Their movements were elegant and composed. The entire zoo was filled with a serene atmosphere.
Then, you visited other exhibits and experienced the entire zoo. After that, you left the zoo.</td></tr>
<tr><td>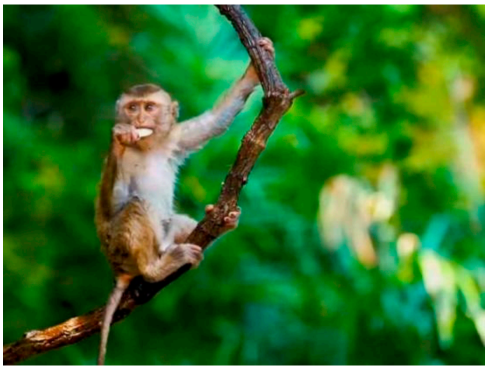</td><td>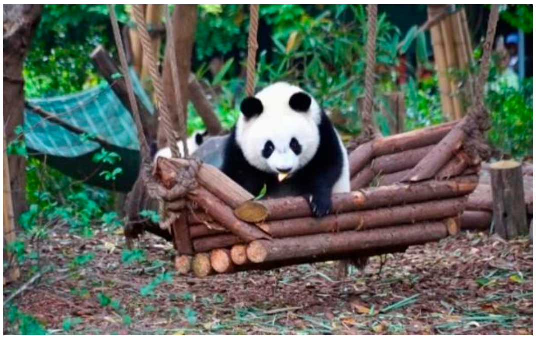</td></tr>
</table>

3. Materials for Experiment 3

3.1 Experimental Materials for Wildlife Anthropomorphism

<table>
<tr><td>Wildlife anthropomorphism</td><td>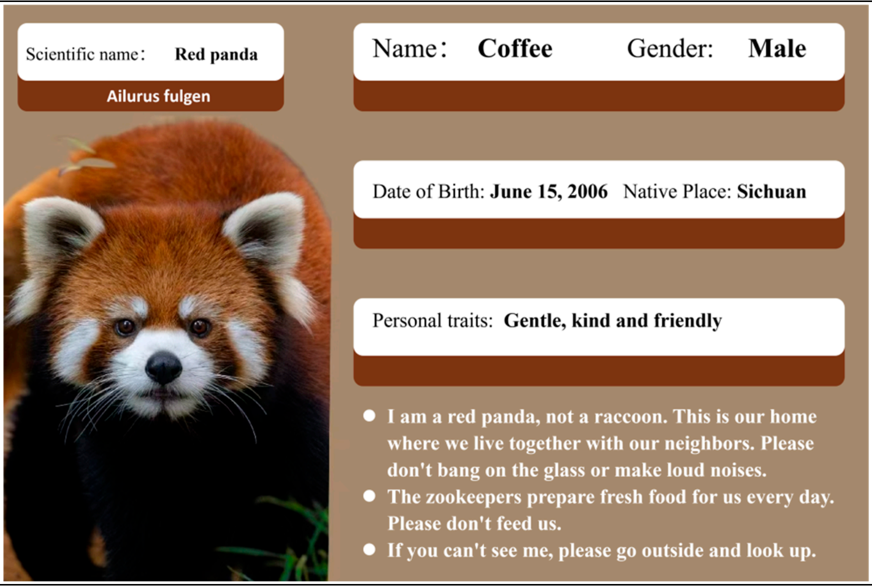
</td></tr>
<tr><td>Non-wildlife anthropomorphism</td><td>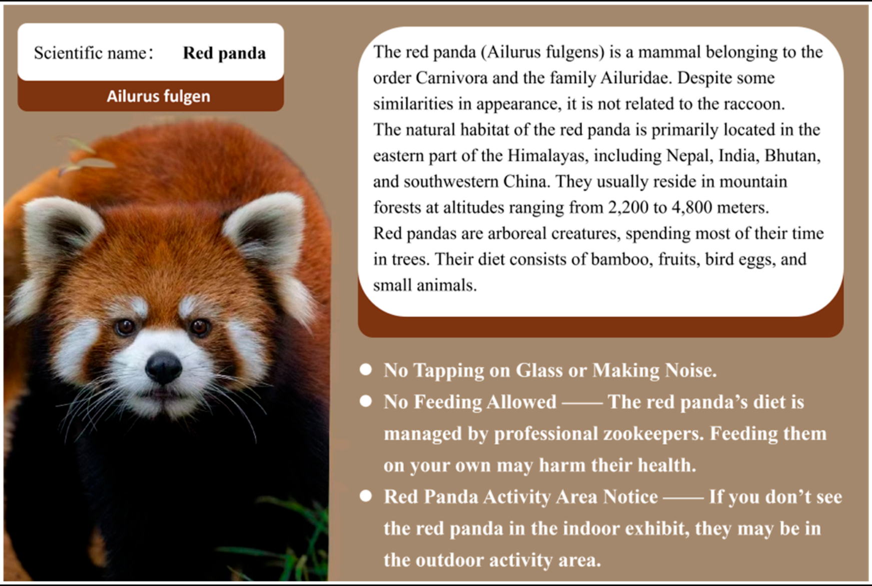
</td></tr>
</table>

Contextual activation	On an early weekend morning, you arrive at the nearby zoo in high spirits, eager to explore its wondrous world.<br>At the crowded entrance, you wait in line to buy a ticket, anticipating the adventure about to unfold. Finally, you receive your pass to this realm of fantasy—the colorful ticket ushers you into this paradise.<br>Once inside, you follow the flow of visitors forward, and before your eyes, various animals appear, each displaying its unique demeanor within its own home.
Positive natural empathy situation（82 words)	Then, you saw the figure of the red panda. The sunlight filtered through the tree branches and fell on the little panda. They were busy rolling on the grass, chasing each other, occasionally gently patting each other with their furry paws, or lazily stretching their bodies and enjoying the warm sunlight. Their eyes shone with curiosity and vitality, and every movement revealed a carefree happiness. Then, you visited other exhibition areas and experienced the entire zoo. After that, you left the zoo.

	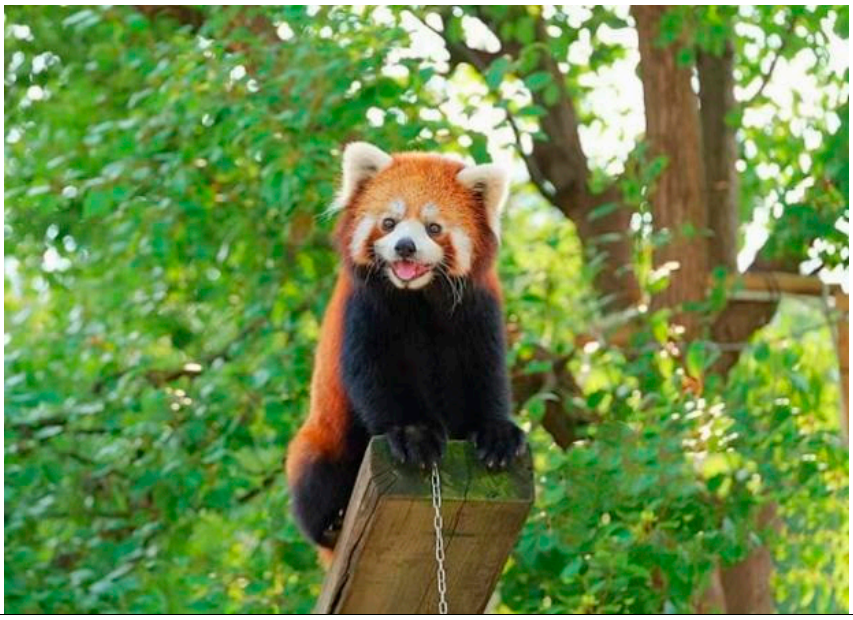
No natural empathy situation (88 words)	Then, you saw the figure of the red panda. The red pandas were either lying leisurely in the shade of the trees or sitting quietly on the rocks. Their eyes were half-closed, enjoying the peacefulness of the afternoon. Occasionally, one of the red pandas would gently wag its tail or slowly chew on the fresh bamboo leaves, moving softly and slowly. The air around was filled with a sense of tranquility. Then, you visited other exhibits and finished the entire zoo experience. After that, you left the zoo.
	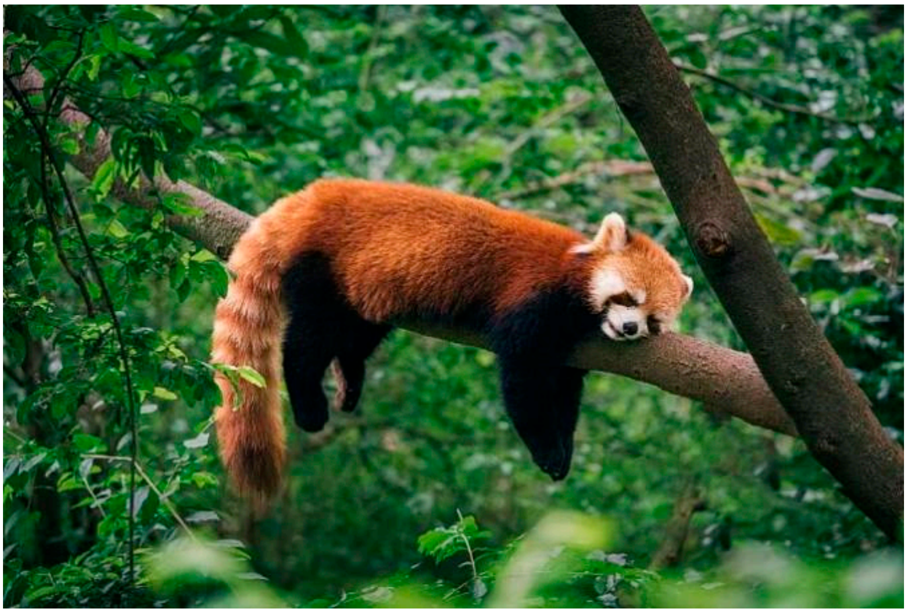
Negative natural empathy situation (85 words)	Then, you saw the figure of the red panda. The red pandas looked listless. They were lying quietly curled up in a corner, their eyes losing the former brilliance, as if shrouded by an invisible haze. Their movements were sluggish, and the low, muffled cries they occasionally emitted seemed to be expressing inner unease and loneliness. There seemed to be a suffocating atmosphere in the surrounding air. Then, you visited other exhibition areas and finished experiencing the entire zoo. After that, you left the zoo.

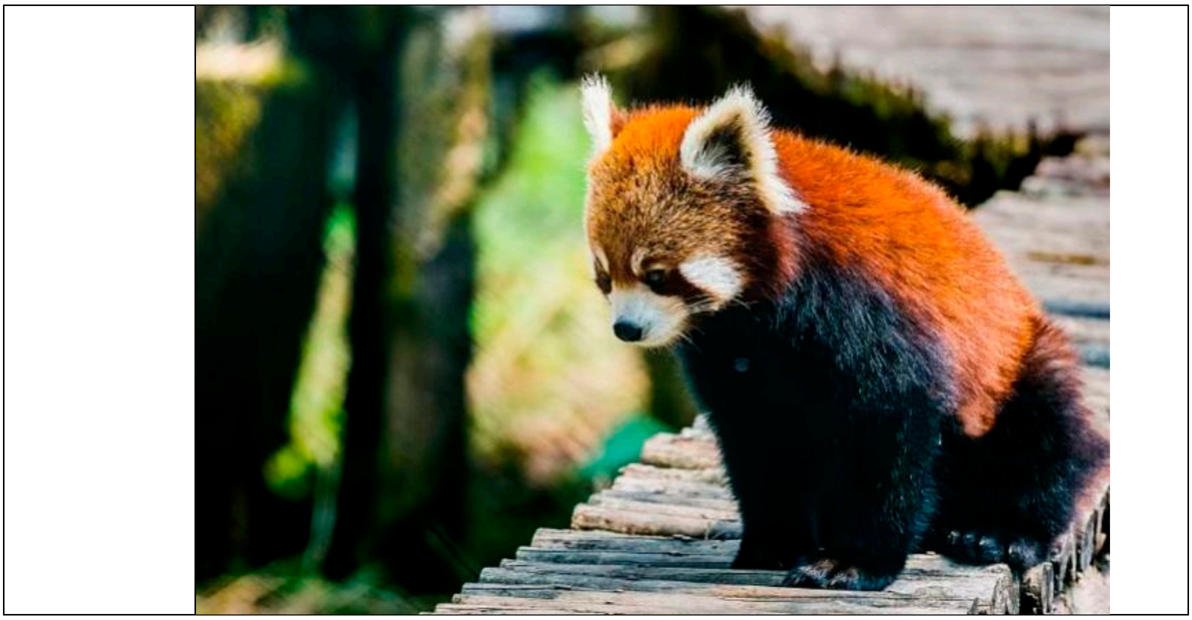

Supplementary Material S3: Questionnaire Scales

The positive and negative empathy scales share four trait-empathy items, which index the dispositional component of empathy and are included in both scales by design; each scale additionally contains four valence-specific items. Each empathy scale thus comprises eight items in total. Horizontal lines separate the item blocks.

Table S1. Trait-empathy items (shared by both the positive and the negative empathy scales; adapted from Andreychik & Migliaccio, 2015; Tam, 2013)

No.	Item
1	I can immerse myself in the feelings of these wildlife.
2	I feel as if I am one of these wildlife.
3	I can easily put myself in the position of these wildlife.
4	I can understand their feelings from these wildlife's perspective.

Table S2. Positive-valence items (administered as part of the positive empathy scale; adapted from Andreychik & Migliaccio, 2015; Tam, 2013)

No.	Item
5	In my mind, I can clearly envision the positive situation of these wildlife.
6	I will envy these wildlife.[1]
7	I can feel the happiness these wildlife experience.
8	I will be happy for these wildlife.

[1] The Chinese item uses 羡慕 (xiànmù), which denotes benign, admiring envy—"I admire them and wish I could share in their joy"—rather than malicious envy; the item is intended to capture the admiring component of empathic joy.

Table S3. Negative-valence items (administered as part of the negative empathy scale; adapted from Andreychik & Migliaccio, 2015; Tam, 2013)

No.	Item
9	In my mind, I can clearly envision the difficult situation of these wildlife.
10	I will be concerned about these wildlife.
11	I can feel the pain these wildlife have suffered.
12	I will sympathize with these wildlife.

Table S4. Restorative experience (Korpela et al., 2008)

No.	Item
1	I feel calmer after being here.
2	After visiting this place I feel restored and relaxed.
3	I get new enthusiasm and energy for my everyday routines from here.
4	My concentration and alertness clearly increase here.
5	I can forget everyday worries here.
6	Visiting here is a way of clearing and clarifying my thoughts.

Table S5. Hedonic well-being (Park & Ahn, 2022)

No.	Item
1	When I was engaged in this activity, I felt more satisfied than when I was involved in most other activities.
2	This tour gives me my strongest sense of enjoyment.
3	When I engage in this tour, I feel good.
4	This activity gave me a lot of pleasure.
5	When I was engaged in this activity, I felt happier than when I was engaged in most other activities.
6	When I engage in this tour, I feel a warm glow.

Table S6. Eudaimonic well-being (Park & Ahn, 2022)

No.	Item
1	This tour gives me my greatest feeling of really being alive.
2	When I engage in this tour I feel more intensely involved than I do when engaged in most other tours.
3	This tour gives me my strongest feeling that this is who I really am.
4	When I engaged in this tour, I felt that this was what I was meant to do.
5	I feel more complete or fulfilled when engaging in this tour than I do when engaged in most other tours.
6	I feel a special fit or meshing when engaging in this tour.

Psychometric validation. Because the two empathy scales share four trait items, a two-factor model over the 16 listed indicators would misrepresent the design; we therefore estimated a three-factor confirmatory factor model on the 12 unique items (trait; positive valence; negative valence) in Experiment 3 (N = 299). The model fit the data well, $\chi^2(51) = 141.85$, $\chi^2/df = 2.78$, CFI = .963, TLI = .952, RMSEA = .077, SRMR = .046; standardized loadings ranged from .64 to .91 (all ps < .001), and CR (.81–.94) and AVE (.52–.78) exceeded conventional thresholds. The positive- and negative-empathy factors remained clearly distinct, r = −.59, p < .001, and the

Fornell–Larcker criterion was satisfied (√AVE .72–.89 exceeding all inter-factor correlations). A one-factor alternative model fit poorly, $\chi^2(54) = 1024.08$, CFI = .604, RMSEA = .246, SRMR = .191, and was rejected, $\Delta\chi^2(3) = 882.23$, $p < .001$.
